# Development, Evaluation, and Implementation of a House-Made Targeted Next-Generation Sequencing Spoligotyping in a French Laboratory

**DOI:** 10.3390/ijms231911302

**Published:** 2022-09-25

**Authors:** Charlotte Genestet, Yannick Baffert, Maxime Vallée, Albin Bernard, Yvonne Benito, Gérard Lina, Elisabeth Hodille, Oana Dumitrescu

**Affiliations:** 1 CIRI—Centre International de Recherche en Infectiologie, Ecole Normale Supérieure de Lyon, Université Claude Bernard Lyon-1, Inserm U1111, CNRS UMR5308, 69007 Lyon, France; 2 Laboratoire de Bactériologie, Institut des Agents Infectieux, Hospices Civils de Lyon, CEDEX 04, 69317 Lyon, France

**Keywords:** tuberculosis, *Mycobacterium tuberculosis* complex, spoligotyping, membrane-based spoligotyping, in silico spoligotyping, CRISPR locus diversity, targeted next-generation sequencing

## Abstract

Epidemiological studies investigating transmission chains of tuberculosis are undertaken worldwide to tackle its spread. CRISPR locus diversity, called spoligotyping, is a widely used genotyping assay for *Mycobacterium tuberculosis* complex (MTBC) characterization. Herein, we developed a house-made targeted next-generation sequencing (tNGS) spoligotyping, and compared its outputs with those of membrane-based spoligotyping. A total of 144 clinical MTBC strains were retrospectively selected to be representative of the local epidemiology. Data analysis of a training set allowed for the setting of “presence”/“absence” thresholds for each spacer to maximize the sensibility and specificity related to the membrane-based spoligotyping. The thresholds above, in which the spacer was considered present, were 50 read per millions for spacers 10 and 14, 20,000 for spacers 20, 21, and 31, and 1000 for the other spacers. The confirmation of these thresholds was performed using a validation set. The overall agreement on the training and validation sets was 97.5% and 93.8%, respectively. The discrepancies concerned six strains: Two for spacer 14, two for spacer 31, and two for spacer 32. The tNGS spoligotyping, whose thresholds were finely-tuned during a careful bioinformatics pipeline development process, appears be a technique that is reliable, inexpensive, free of handling errors, and automatable through automatic transfer into the laboratory computer system.

## 1. Introduction

The control of *Mycobacterium tuberculosis* complex (MTBC) transmission in high-income and low-tuberculosis (TB) prevalence countries remains a public health priority considering the constant changes in MTBC epidemiology worldwide. Key measures for TB control rely on the linkage of cases and on the identification of transmission chains, performed through a population-based systematic molecular TB survey to uncover outbreaks, even between apparently unrelated cases [1,2]. Therefore, regional reference laboratories mastering MTBC genotyping techniques are needed as they would allow for a territorial coverage.

The analysis of clustered regulatory interspaced short palindromic repeats (CRISPR) locus diversity, called spoligotyping, is a widely used genotyping assay for the characterization of MTBC and epidemiological purposes [3,4]. Historically, spoligotyping detected the presence or absence of 43 unique spacers in the direct repeat (DR) region of the CRISPR locus of MTBC. This was based on an initial PCR using primers amplifying the most frequently occurring DR sequences, followed by a reverse line blot hybridization membrane-based revelation method [5,6]. This membrane-based method is laborious and not automatable, and relies on the steps of manual analysis that can be a source of errors, but it is feasible from inactivated bacterial lysates containing low DNA amounts. Since the advent of next generation sequencing (NGS), whole-genome sequencing (WGS) has been implemented in high-income countries and has profoundly transformed the perspectives of TB diagnosis. WGS provides a better discriminatory power than spoligotyping to determine the relatedness between MTBC isolates [7]. In addition, WGS allows for the attainment of quick and accurate genotypic antimicrobial susceptibility testing and MTBC species identification using single nucleotide polymorphism (SNP) without prior specific PCR amplification [8]. Moreover, several pipelines have been developed to extract a spoligotype from WGS data, also called in silico spoligotyping [9], enabling a continuity of MTBC molecular surveys. Previously, it has been shown that a smooth transition from the membrane-based to the in silico WGS-based genotyping of MTBC isolates was possible for TB diagnosis and epidemiological survey despite discrepancies with the membrane-based method, notably related to the insertion of sequence (IS) 6110 in the DR region flanking the spacer 31 [10].

Nevertheless, in silico WGS-based spoligotyping requires large amounts of pure mycobacterial DNA extract. For proximity laboratories unable/unwilling to provide high inoculum MTBC cultures (requiring BSL3 facilities and costly transportation), it was necessary to develop an in silico spoligotyping technique compatible with inactivated samples containing low DNA amounts. A new commercial in vitro diagnostic-targeted NGS test, the Deeplex Myc-TB (GenoScreen, Lille, France), allows for the targeting of 18 MTBC drug resistance-associated genes, combined with genomic targets for mycobacterial species identification and MTBC spoligotyping from samples containing low DNA amounts [11,12]. However, this test remains expensive and no comparison of the outputs of these different methods is available.

Herein, we developed and routinely implemented a house-made targeted next-generation sequencing (tNGS) spoligotyping method after a PCR amplification step of the DR locus, feasible from inactivated bacterial lysates containing low DNA amounts. This assay was compared with both the membrane-based method and the in silico WGS-based spoligotyping in terms of output, allowing for a continuity of MTBC molecular surveys at the regional level in a cost-effective manner.

## 2. Results

### 2.1. MTBC Isolates

**Figure 1:**
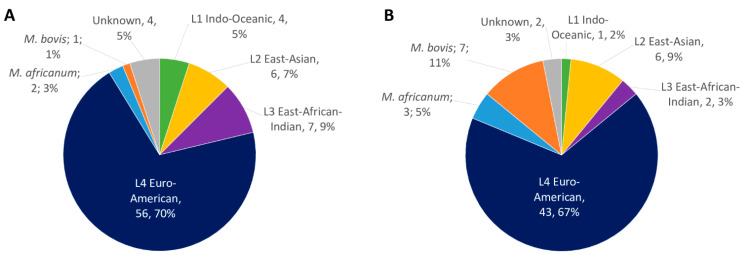
Epidemiology of 144 MTBC strains included in the study, separated on (**A**) training set (*n* = 80 strains) and (**B**) validation set (*n* = 64 strains). For both, the identification was based on membrane-based spoligotyping identification.

Between January 2017 and February 2018, 144 MTBC isolates from specimens sampled from patients during routine care in the Hospices Civils de Lyon, France were retrospectively selected to be representative of the local epidemiology. MTBC strains were separated in two homogenous sets (in terms of lineage diversity, both representing the local epidemiology (Genestet et al. 2022 [10]; Barbier et al. 2018 [13])): A training set (80 strains) and a validation set (64 strains; Figure 1).

MTBC spoligoytpes were determined by two methods: Membrane-based spoligotyping and a house-made tNGS spoligotyping, including an initial PCR amplification step of the DR locus.

### 2.2. Identification of Thresholds Defining the “Presence” or “Absence” of Spacers

**Figure 2:**
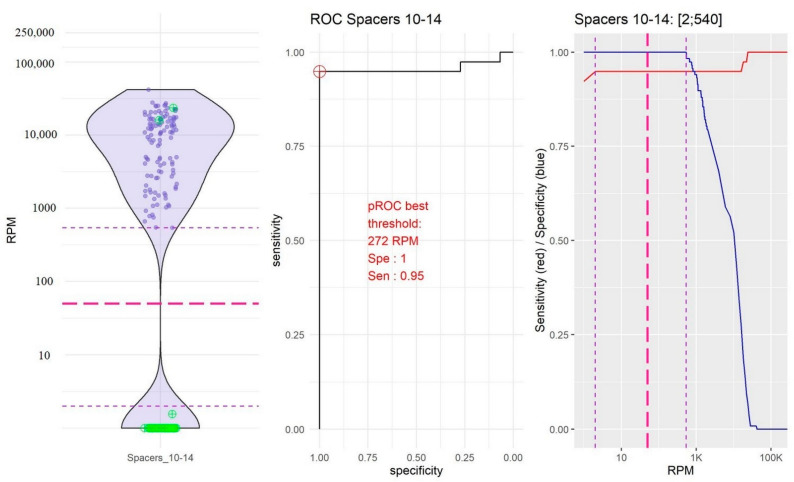
Determination of the reads per million (RPM) threshold for spacers 10 and 14. On the left: Distribution in the form of a violin plot of spacers 10 and 14 RPM. Purple circles represent the “present” spacers according to the membrane-based spoligotyping. Green circles represent the “absent” spacers according to the membrane-based spoligotyping. Dotted red line: RPM threshold choice (50 RPM). In the middle: Receiver operating characteristic (ROC) curve for spacers 10 and 14. Red circle represents the best RPM threshold. On the right: Sensibility (red line) and specificity (blue line) compared to the membrane-based spoligotyping according to the variable RPM threshold. Dotted purple line: RPM thresholds between which the sensitivity and the specificity are maximized. Dotted red line: RPM threshold choice (50 RPM).

**Figure 3:**
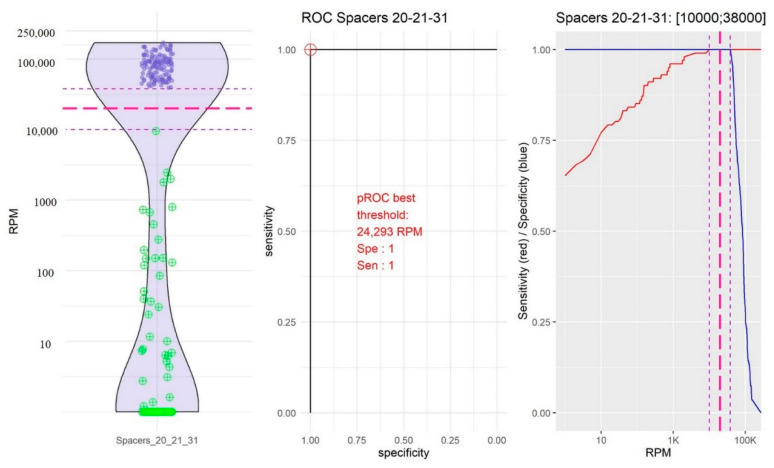
Determination of the reads per million (RPM) threshold for spacers 20, 21, and 31. On the left: Distribution in the form of a violin plot of spacers 20, 21, and 31 RPM. Purple circles represent the “present” spacers according to the membrane-based spoligotyping. Green circles represent the “absent” spacers according to the membrane-based spoligotyping. Dotted red line: RPM threshold choice (20,000 RPM). In the middle: Receiver operating characteristic (ROC) curve for spacers 20, 21, and 31. Red circle represents the best RPM threshold. On the right: Sensibility (red line) and specificity (blue line) compared to the membrane-based spoligotyping according to the variable RPM threshold. Dotted purple line: RPM thresholds between which the sensitivity and the specificity are maximized. Dotted red line: RPM threshold choice (20,000 RPM).

**Figure 4:**
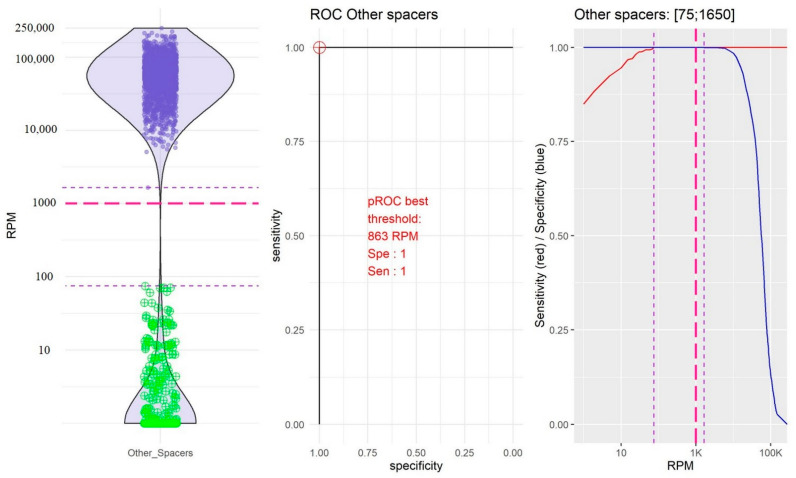
Determination of the reads per million (RPM) threshold for the other spacers. On the left: Distribution in the form of a violin plot of the other spacers. Purple circles represent the “present” spacers according to the membrane-based spoligotyping. Green circles represent the “absent” spacers according to the membrane-based spoligotyping. Dotted red line: RPM threshold choice (1000 RPM). In the middle: Receiver operating characteristic (ROC) curve for the other spacers. Red circle represents the best RPM threshold. On the right: Sensibility (red line) and specificity (blue line) compared to the membrane-based spoligotyping according to the variable RPM threshold. Dotted purple line: RPM thresholds between which the sensitivity and the specificity are maximized. Dotted red line: RPM threshold choice (1000 RPM).

As bioinformatics analyses were performed from amplified sequences, the definition of thresholds to validate the “presence” or “absence” of spacers constituted a crucial step in the development of the pipeline, notably to avoid false positive spacers. From the training dataset, a violin plot, a ROC curve, and a plot of the sensibility and specificity according to the thresholds were generated for each spacer using membrane-based spoligotyping as the reference method (Supplementary Figures S1–S43). According to their distribution, three groups of spacers could be distinguished:

- The group of spacers 10 and 14 in which the RPM mean value of “present” spacers was the lowest (10,754.39, 95% confidence interval, CI [9251.10; 12,257.67]) and the RPM mean value of “absent” spacers was very low (0.04, 95% CI [−0.04; 0.18]; Figure 2).
- The group of spacers 20, 21, and 31 in which the RPM mean value of “present” spacers was high (84,941.72, 95% CI [79,478.97; 90,404.48]) and the RPM mean value of “absent” spacers was the highest (192.39, 95% CI [2.437339; 382.353323]; Figure 3).
- The group of the other spacers in which the RPM mean value of “present” spacers was high (61,400.65, 95% CI [59,776.49; 63,024.80]) and the RPM mean value of “absent” spacers was low (11.45; 95% CI [4.14; 18.76]; Figure 4).

After data analysis of the three groups, the thresholds above in which the spacer was considered present were 50 RPM for spacers 10 and 14, 20,000 RPM for spacers 20, 21, and 31, and 1000 RPM for the other spacers.

### 2.3. Validation of the Defined Thresholds and Concordance between Membrane-Based Spoligotyping and tNGS Spoligotyping

According to the thresholds defined above, the overall agreements on the training set were 97.5% (78/80; 95% CI [91.3; 99.7]) at the sample level and 99.9% (3438/3440; 95% CI [99.8; 99.9]) at the spacer level. The discrepancies concerned two strains, and each time spacer 14 was “absent” with the membrane-based method and “present” with tNGS spoligotyping. For this spacer, the Cohen’s Kappa coefficient indicated that the concordance between membrane-based spoligotyping and tNGS spoligotyping was illustrative of an almost perfect agreement (Cohen’s Kappa > 0.81; Table 1).

The data of the validation set (64 MTBC strains) were also analyzed according to the previously defined thresholds. The overall agreements on the validation set were 93.8% (60/64; 95% CI [84.8; 98.3]) at the sample level and 99.8% (2748/2752; 95% CI [99.6; 99.9]) at the spacer level. The discrepancies concerned four strains: Two strains for spacer 31 that was “present” with the membrane-based method and “absent” with tNGS spoligotyping, and two strains isolated from the same patient for spacer 32 that was “absent” with the membrane-based method and “present” with tNGS spoligotyping. For these spacers, Cohen’s Kappa coefficient indicated that the concordance between membrane-based spoligotyping and tNGS spoligotyping was illustrative of an almost perfect agreement (Cohen’s Kappa > 0.81; Table 1).

After pooling the training and validation sets, the overall agreements were 95.8% (138/144; 95% CI [91.2; 98.5]) at the sample level and 99.9% (6186/6192; 95% CI [99.8; 99.9]) at the spacer level. Of note, these discrepancies had no impact on the identification of MTBC species or of *M. tuberculosis* lineage.

### 2.4. Discrepancy Analysis between Membrane-Based, tNGS, and In Silico Spoligotyping

**Figure 5:**
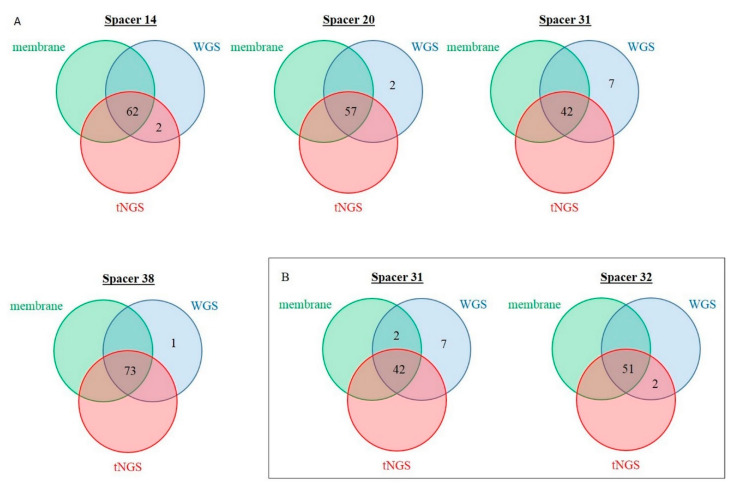
Representation of “present” discordant spacers using Venn diagrams for membrane- (green), targeted-next generation sequencing (tNGS; red), and whole genome sequencing (WGS; blue) based spoligotyping: (**A**) For the training set, (**B**) for the validation set.

To investigate the discrepancies between membrane-based spoligotyping and tNGS spoligotyping, in silico spoligotyping using WGS was generated for 144 MTBC strains, allowing for a reconstruction of the CRISPR locus in MTBC using CRISPR-builder TB [14]. The discrepancies between the three methods were reported in Venn diagrams (Figure 5, Supplementary Material Table S1).

Overall, spacer 31 was the spacer related to most of the discordance between in silico spoligotyping and the other methods based on the initial PCR.

Concerning the two discrepant MTBC isolates between membrane-based and tNGS spoligotyping of the training set (spacer 14, “absent” with the membrane-based method and “present” with tNGS spoligotyping), the in silico spoligotyping was concordant with the tNGS method. The reconstruction of the CRISPR locus using CRISPR-builder TB did not find any genetic element that could explain the discrepancies. Concerning the two discrepant MTBC isolates on spacer 31 between membrane-based and tNGS spoligotyping of the validation set (“present” with the membrane-based method and “absent” with tNGS spoligotyping), the in silico spoligotyping was concordant with the membrane-based method. For these MTBC isolates, CRISPR-builder TB found an insertion of the insertion sequence (IS) 6110 within the DR sequence upstream the spacer 31 for one strain, and within the DR sequence downstream the spacer 31 for the other strain. Concerning the two discrepant MTBC isolates on the spacer 32 between membrane-based and tNGS spoligotyping of the validation set (“absent” with the membrane-based method and “present” with tNGS spoligotyping), the in silico spoligotyping was concordant with the tNGS method. For these MTBC isolates, CRISPR-builder TB found an insertion of the IS6110 within the DR sequence upstream of spacer 32 for both strains.

## 3. Discussion

In the present study, the finely-tuned development of the “house-made” bioinformatics pipeline was a key step in the development of tNGS spoligotyping. This method includes an initial PCR step for the amplification of the spacer sequence. In addition, the spacer “presence”/“absence” thresholds should be different from a technique without amplification, e.g., in silico spoligotyping using WGS, to avoid the risk of false positive results related to inter-sample contaminations. Therefore, NGS data analysis using free online pipelines, such as SpoTyping [9], was impossible and would have yielded erroneous results. This is why the threshold above, in which the spacers were defined as “present” for the tNGS was high (1000 RPM) for the majority of the spacers. Concerning the lower threshold for spacers 10 and 14 (50 RPM), no genomic explanation was found. Concerning the higher threshold for spacers 20, 30, and 31 (20,000 RPM), the choice of the reference method greatly influenced this threshold. The MTBC CRISPR locus, which is the preferential insertion site for the IS6110 that possibly disrupts DR or adjacent spacer sequences [15] of both DR variations and IS insertion, may hamper primer affinity resulting in incomplete or abortive DNA amplification. These genomic alterations (the insertion of IS6110 within the DR sequence upstream or downstream of the spacers, the presence of mutated DR, or the presence of truncated spacers) modify the expected spoligotype patterns, despite the presence of spacers within the CRISPR locus [10,16,17,18]. As observed herein, the most discordant spacer due to the insertion of IS6110 between the in silico spoligotyping using WGS and the PCR-based spoligotyping (membrane-based method and tNGS spoligotyping) was spacer 31 [10,15,16,17]. This led to a significant change in the local epidemiology of the laboratory during the transition to in silico spoligotyping with WGS, e.g., the disappearance of MTBC strains belonging to the SIT 50. Indeed, it has been previously shown that, using membrane-based spoligotyping, the SIT number was 50 for all MTBC strains (i.e., no detection of spacer 31), whereas using in silico spoligotyping, the SIT number was actually 53 (i.e., with detection of spacer 31) due to the insertion of IS6110 within the DR sequence downstream of spacer 31 [10]. Due to the phylogenetic relevance of this insertion [5,19,20], the classification of the corresponding strains as SIT 50 rather than SIT 53 should be preferred. For the development of tNGS spoligotyping, the elongation time of the PCR was extended (herein at 60 s), thereby allowing for more or less effective amplification of the spacer sequence despite the presence of IS6110 in the DR sequence. To ensure a continuity in the interpretation of spoligotyping results and allow for the identification of SIT 50, we chose the membrane-based method as the reference method. Therefore, for a MTBC strain identified as belonging to SIT 50 with the membrane-based method, even in the case of scarce PCR amplification of spacer 31, the relatively high threshold of tNGS spoligotyping (20,000 RPM for spacer 31) allowed for assigning SIT 50 for this strain as well, whereas the in silico spoligotyping would have assigned SIT 53.

After the finely-tuned development of the “house-made” bioinformatics pipeline to define the appropriate detection thresholds for each of the spacers, the tNGS spoligotyping relying on the “house-made” pipeline analysis had a very good overall agreement with the membrane-based method at the sample level. The overall agreement was significantly higher than the one found between the membrane-based method and the in silico spoligotyping using WGS [10]. The reconstruction of the CRISPR locus using CRISPR-builder TB allowed for the identification of the origin of the observed discrepancies between tNGS spoligotyping and the membrane-based method for four MTBC strains out of six discrepancies. For two MTBC strains, in which spacer 14 was detected by tNGS and confirmed by in silico spoligotyping using WGS, but not detected by the membrane-based method, no genomic genetic phenomenon concerning spacer 14 was found using CRISPR-builder TB. Therefore, we were not able to conclude why the membrane-based method failed to detect this spacer. Another hypothesis, such as a particular DNA conformation of that spacer impacting the amplification step, may explain this failed detection. For two MTBC strains isolated from the same patient, spacer 32 was detected by tNGS spoligotping and confirmed by in silico spoligotyping using WGS, but was not detected by the membrane-based method due to the insertion of IS6110 within the DR sequence upstream of spacer 32. In these cases, the extended elongation time of the tNGS spoligotyping PCR step probably enabled the sufficient amplification of the spacer 32 sequence, coupled with a relatively low threshold of 1000 RPM, overall resulting in the detection of spacer 32 by tNGS spoligotyping. For two MTBC strains, spacer 31 was not detected by tNGS spoligotyping due to the insertion of IS6110 within the DR sequence upstream or downstream of spacer 31. Of note, the membrane-based technique could also be expected to fail in detecting the spacer 31 of these strains. A careful examination of the membrane from this spoligotyping run revealed that the markings of spacers 31 for these two strains were significantly more faded than the markings of the other spacers 31, and that in the end, the result could have been interpreted as the absence of spacers 31. Therefore, these two differences regarding spacer 31 between the tNGS spoligotyping and the membrane-based method could be artefactual, highlighting the fact that the membrane reading is subjective and that a more objective technique, such as tNGS spoligotyping is required to ensure more consistency in the spoligotyping results. Furthermore, unlike the membrane-based method that requires a manual entry of results, as the output values of the tNGS spoligotyping can be generated in .csv format, the results can be transferred directly into the laboratory IT management system through a computer connection.

In terms of reaction cost, tNGS spoligotyping (around EUR 24 per sample) is more low-cost than the membrane-based method (around EUR 30 per sample) or the in silico spoligotyping using WGS (around EUR 28 per sample). Moreover, tNGS spoligotyping has the advantages of requiring less technical staff compared to the membrane-based method, and of being feasible using inactivated bacterial lysates containing few DNA amounts compared to in silico spoligotyping using WGS. The tNGS spoligotyping method could be performed by NGS-equipped national or supranational reference centers in low-income countries, from samples sent by outlying (remote) TB diagnosis centers to ensure a national epidemiological survey. As the diversity of the CRISPR locus has been shown to accurately reflect the phylogeny of MTBC, spoligotyping can be used not only for epidemiological purposes, but also for MTBC species identification using an algorithmic approach in routine TB diagnosis [21]. Consequently, tNGS spoligotyping can also be used as a low-effective method of MTBC species identification.

## 4. Materials and Methods

### 4.1. MTBC Conventional Spoligotyping

Membrane-based spoligotyping experiments were performed as described elsewhere [5]. MTBC spoligotyping-based identification and the shared international type (SIT) number determination were provided through the open access SITVITWEB [22] and SpolLineages software tool, https://github.com/dcouvin/SpolLineage (accessed on 13 March 2021) [21].

### 4.2. MTBC DR Locus tNGS

The first step PCR was directly performed on warm inactivated mycobacterial lysates without preliminary DNA extraction. Briefly, the PCR mix contained the same primers as those used for membrane-based spoligotyping, except for being biotin-free (DRa 5′GGTTTTGGGTCTGACGAC-3′; DRb 5′-CCGAGAGGGGACGGAAAC-3′) and a Platinum™ SuperFi™ polymerase (Invitrogen, Waltham, MA, USA). The PCR reaction was performed in a thermal cycler peqSTAR (peqQtar Doppio, Ozyme, Saint-Cyr l’Ecole, France) according to the following program: 1 cycle of DNA denaturation at 98 °C for 30 s; 30 cycles of amplification at 98 °C for 10 s (denaturation); 55 °C for 10 s (hybridization); 72 °C for 60 s (elongation); and 1 cycle at 72 °C for 5 min. The obtained amplicons were diluted twice in PCR-grade water and stored at +4 °C until sequencing.

Libraries were generated using a bead-based tagmentation system (DNAprep; Illumina, San Diego, CA, USA). A nanoliter liquid handler (mosquito HV; SPTLabtech, Hertfordshire, UK) was used to reduce the reaction volumes by 10 times. Miniaturized libraries were sequenced on the Nextseq system (Illumina) to produce 150 base-pair paired-end reads. A minimum number of sequenced reads of 100,000 reads was obtained for each isolate.

### 4.3. tNGS Spoligotyping

To ensure a continuity in the interpretation of spoligotyping results, the membrane-based method used for 30 years was considered as the reference method for data analysis and choice of the tNGS threshold. For this purpose, a “house-made” pipeline SpoMutScan was developed. SpoMutScan code leverages MutScan capabilities [23], coupled with GNU parallel [24]. For reproducibility purposes, especially in a routine laboratory, the Shell script was embedded in a Singularity image. The code is freely available at https://gitlab.inria.fr/HCL/software/spomutscan (accessed on 13 March 2021).

A list of 43 strings, corresponding to the 43 spacers, was included in the image. Those strings were prepared to be in the format adapted to MutScan as input. A total of 43 MutScan commands were then generated, one for each spacer. MutScan outputted the raw number of reads containing the targeted strings. SpoMutScan counted and maintained the total number of reads presented as input. Each raw count for the spacers was then normalized against the total number of sequenced reads and multiplied by 1 million (similar to the read per million [RPM] normalization in transcriptomic studies). The purpose of this normalization was to set up RPM thresholds independent from the sequencing depth of samples. Eventually, the SpoMutScan generated binary spoligotypes regarding the previously mentioned RPM thresholds (different spacers requiring different thresholds). From binary spoligotypes, octal spoligotypes were derived. SpoMutScan outputted two files: A log file containing normalized counts for each spacer, and a file with binary and octal spoligotypes.

### 4.4. MTBC In Silico WGS-Based Spoligotyping

For MTBC WGS, genomic DNA was purified from cleared lysates using the Maxwell RSC Instrument (Promega, Madison, WI, USA) automated DNA extraction system and the Maxwell RSC Blood DNA Kit (Promega). Libraries were generated using a bead-based tagmentation system (DNAprep; Illumina, San Diego, CA, USA). A nanoliter liquid handler (mosquito HV; SPTLabtech, Hertfordshire, UK) was used to reduce the reaction volumes by 10 times. Miniaturized libraries were sequenced on the Nextseq or Miseq system (Illumina) to produce 150 or 300 base-pair paired-end reads, respectively. Reference genome coverage was at least 96% and the depth of coverage at least 30×.

MTBC in silico spoligotypes were determined through the open access tool SpoTyping (https://github.com/xiaeryu/SpoTyping-v2.0; accessed on 13 March 2021) [9].

### 4.5. Discrepancy Analysis

To identify the potential events responsible for discrepancies between membrane-based and tNGS spoligotyping (e.g., a spacer found absent “0” in membrane-based but present “1” in tNGS spoligotyping or vice versa), MTBC WGS was performed and allowed for the attainment of in silico spoligotyping and the reconstruction of CRISPR locus using CRISPR-builder TB (https://github.com/cguyeux/CRISPRbuilder-TB, accessed on 13 March 2021) [14].

### 4.6. Data Analysis

Violin plots, receiver operating characteristic (ROC) curves, and plots of sensibility/specificity according to the thresholds were built using R (R Core Team (2018). R is a language and environment for statistical computing (R Foundation for Statistical Computing, Vienna, Austria). Cohen’s Kappa values were calculated using XLSTAT 2020.5.1 (Addinsoft, Paris, France) and interpreted according to Landis and Koch criteria [25].

## 5. Conclusions

In conclusion, tNGS spoligotyping is a reliable spoligotyping method, feasible from inactivated bacterial lysates containing low DNA amounts, free of handling errors, automatable through automatic transfer into the laboratory computer system, and allow for a continuity of MTBC molecular surveys at the regional level in a cost-effective manner. Therefore, this technique has been implemented as a routine in the mycobacterial laboratory of the *Hospices Civils de Lyon* since April 2022.

## Figures and Tables

**Table 1:** Discordant spacers between membrane-based spoligotyping and tNGS spoligotyping for the training set and the validation set.

	Discordant Spacer (Number)	Prevalence in Membrane-Based Spoligotyping, n	Number of Concerned Isolates	Overall Agreement, %	Cohen’s Kappa
**Training set**					
“0” in membrane, “1” in tNGS	14	62	2	97.5	0.911
**Validation set**					
“0” in membrane, “1” in tNGS	32	51	2	96.9	0.898
“1” in membrane, “0” in tNGS	31	44	2	96.9	0.929

“0” corresponds to the absence of the spacer; “1” corresponds to the presence of the spacer. NGS, next-generation sequencing.

## Data Availability

The data presented in this study are available on request from the corresponding author.

## Supplementary Materials

The following supporting information can be downloaded at: https://www.mdpi.com/article/10.3390/ijms231911302/s1.
