# Supplementary material for: Development, Evaluation, and Implementation of a House-Made Targeted Next-Generation Sequencing Spoligotyping in a French Laboratory
Source: Int J Mol Sci. 2022 Sep 25;23(19):11302. doi: 10.3390/ijms231911302 (PMC9569608; doi:10.3390/ijms231911302)
Supplement: Supplementary file 1 [file ijms-23-11302-s001.zip › Supplementary online figures.pdf]

Figure S1 – S43: On the left: a violin plot. Purple circles represent the "present" spacers according to the membrane-based spoligotyping. Green circles represent the "absent" spacers according to the membrane-based spoligotyping. In the middle: Receiver operating characteristic (ROC) curve. Red circle represents the best RPM threshold. On the right: Sensibility (red line) and specificity (blue line) compared to the membrane-based spoligotyping according to the variable RPM threshold. Dotted purple line: RPM thresholds between which the sensitivity and the specificity are maximized.

Figure S1

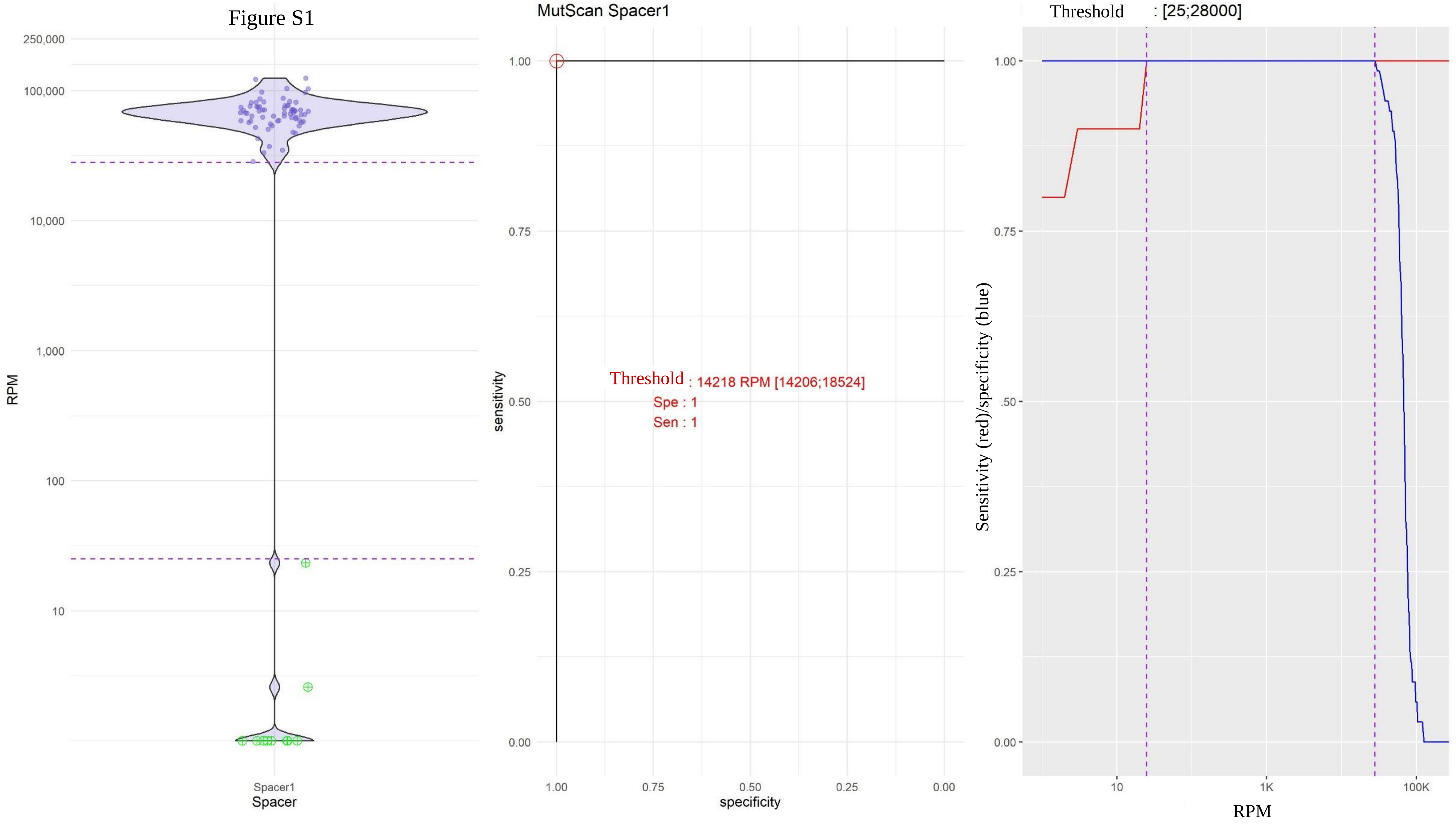

250,000
100,000
10,000
1,000
100
10
RPM
Spacer1
Spacer
MutScan Spacer1
1.00
0.75
0.50
0.25
0.00
sensitivity
specificity
Threshold : 14218 RPM [14206;18524]
Spe : 1
Sen : 1
Threshold : [25;28000]
Sensitivity (red)/specificity (blue)
10
1K
100K
RPM

Figure S2

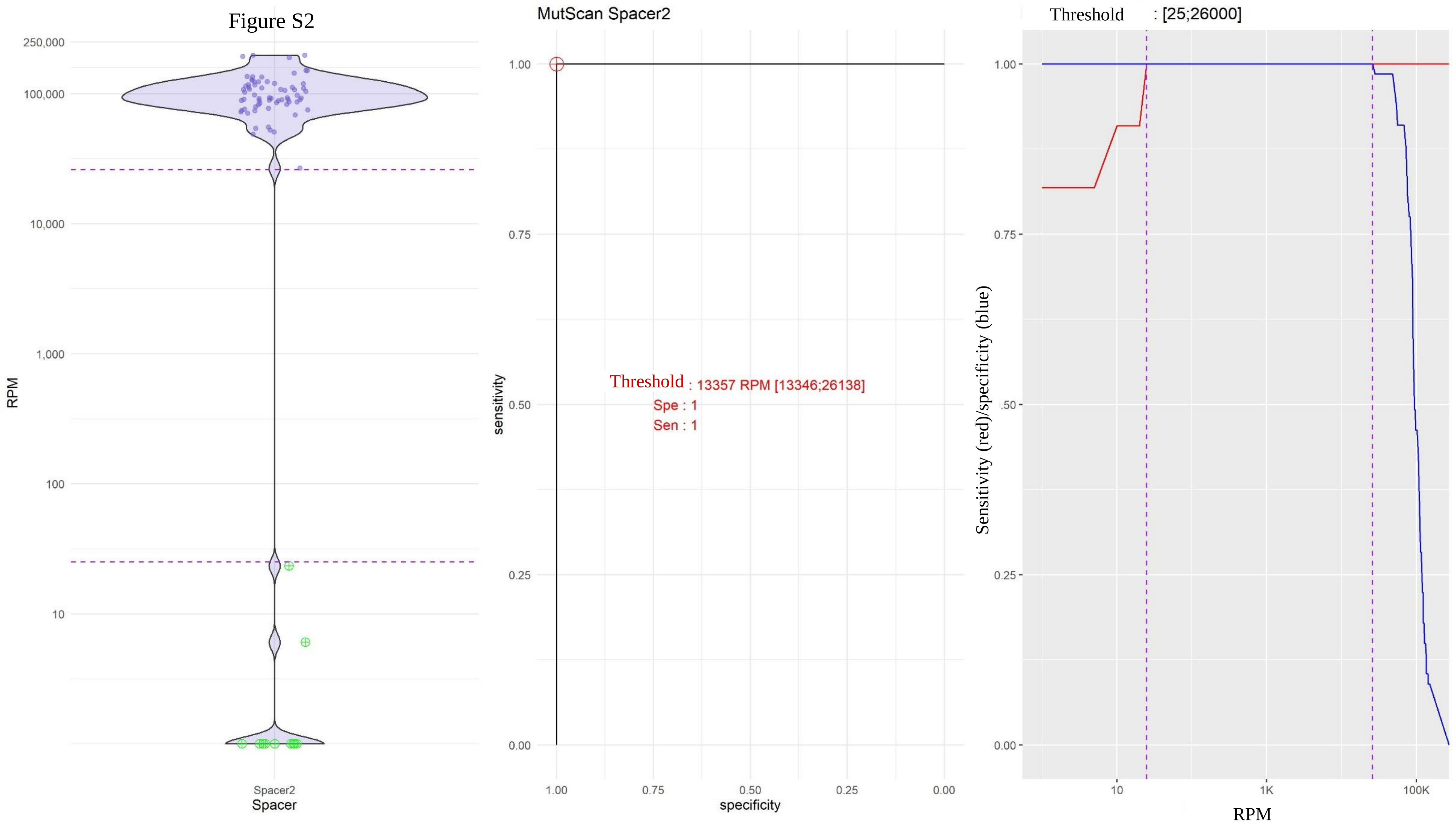
250,000
100,000
10,000
1,000
100
10
RPM
Spacer2
Spacer
MutScan Spacer2
1.00
0.75
0.50
0.25
0.00
sensitivity
specificity
Threshold : 13357 RPM [13346;26138]
Spe : 1
Sen : 1
Threshold : [25;26000]
Sensitivity (red)/specificity (blue)
10
1K
100K
RPM

Figure S3

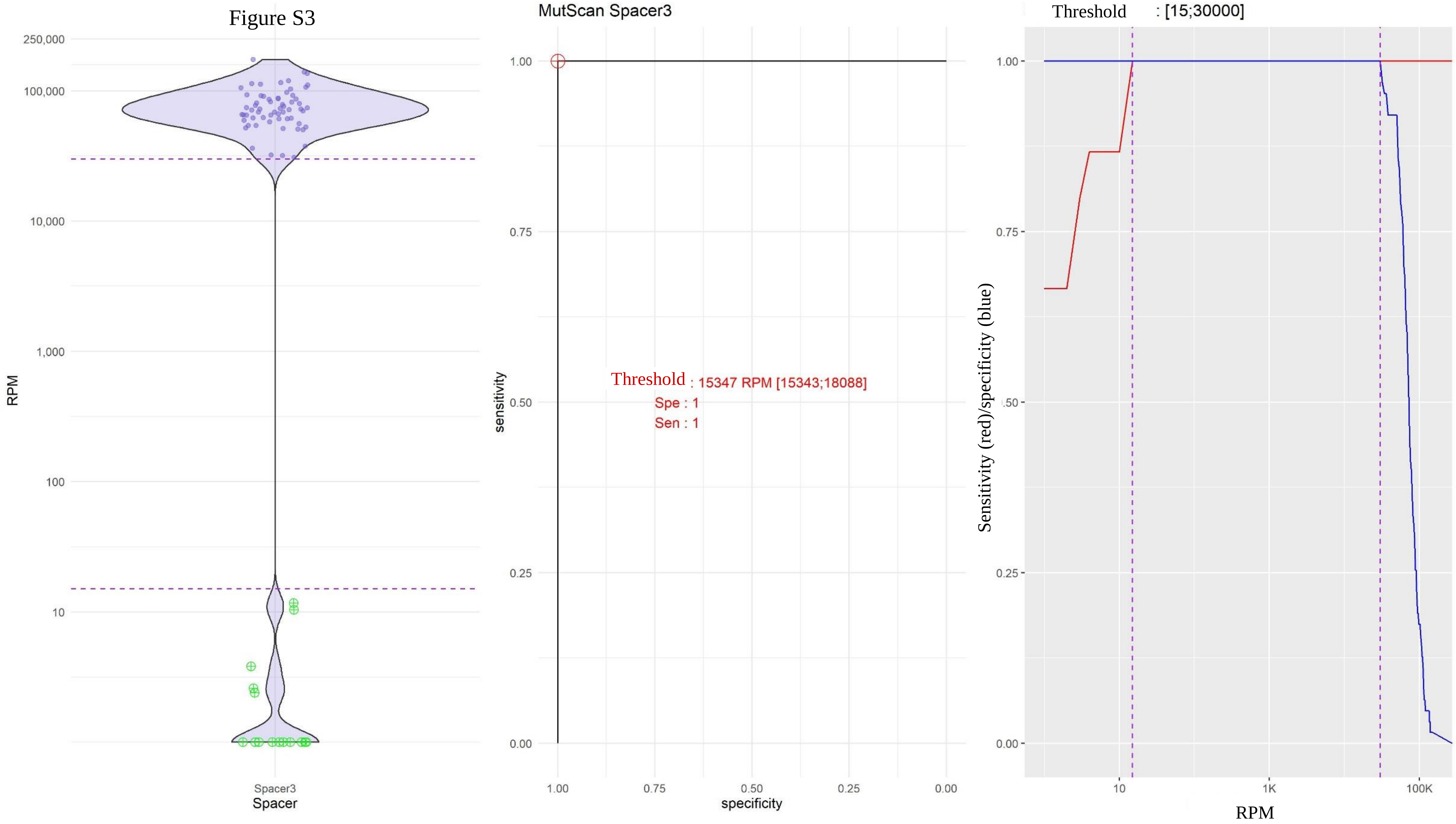

250,000
100,000
10,000
1,000
100
10
RPM
Spacer3
Spacer
MutScan Spacer3
1.00
0.75
0.50
0.25
0.00
sensitivity
specificity
Threshold : 15347 RPM [15343;18088]
Spe : 1
Sen : 1
Threshold : [15;30000]
Sensitivity (red)/specificity (blue)
10
1K
100K
RPM

Figure S4

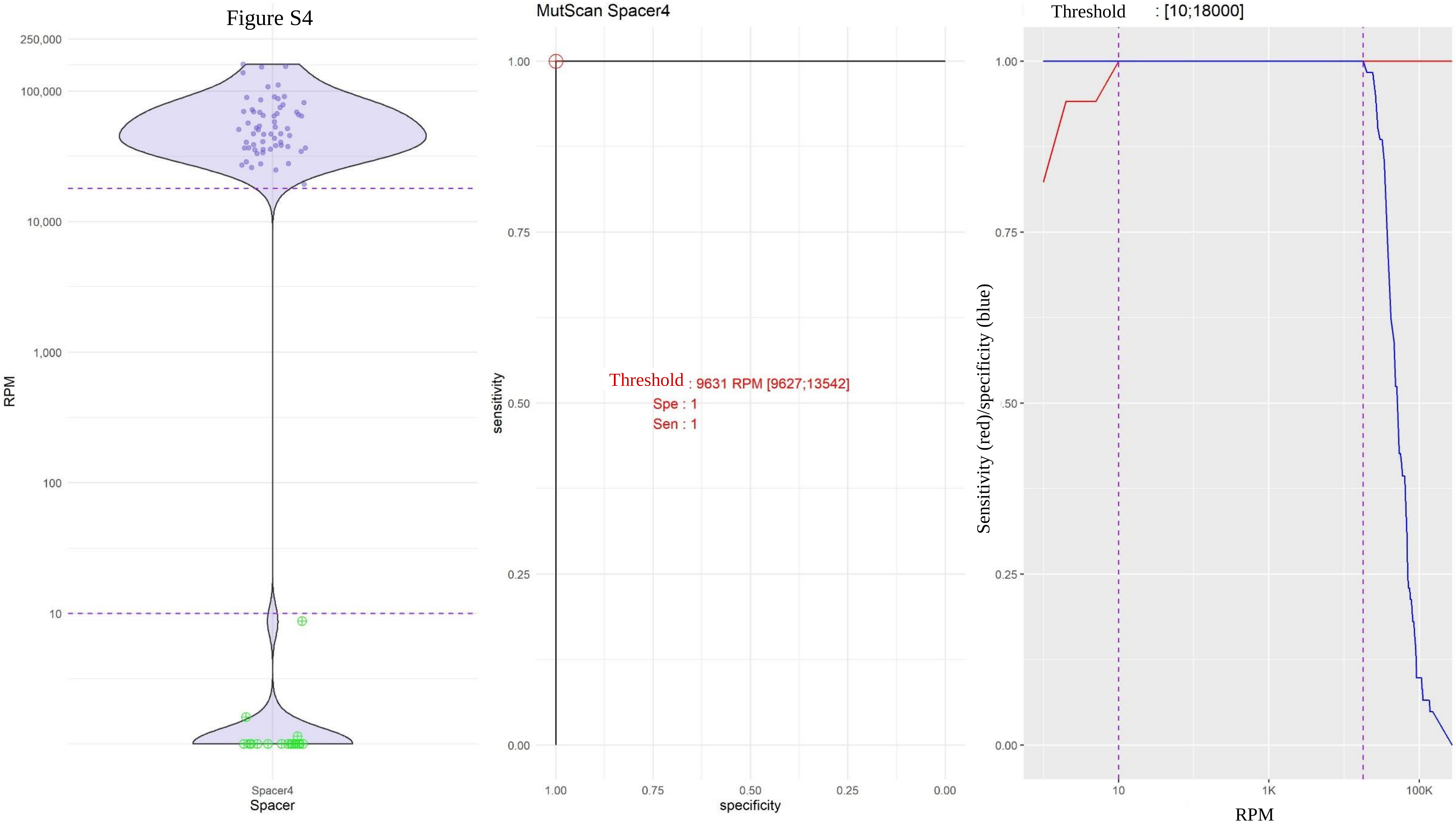
250,000
100,000
10,000
1,000
100
10
RPM
Spacer4
Spacer
MutScan Spacer4
1.00
0.75
0.50
0.25
0.00
sensitivity
specificity
Threshold : 9631 RPM [9627;13542]
Spe : 1
Sen : 1
Threshold : [10;18000]
Sensitivity (red)/specificity (blue)
10
1K
100K
RPM

Figure S5

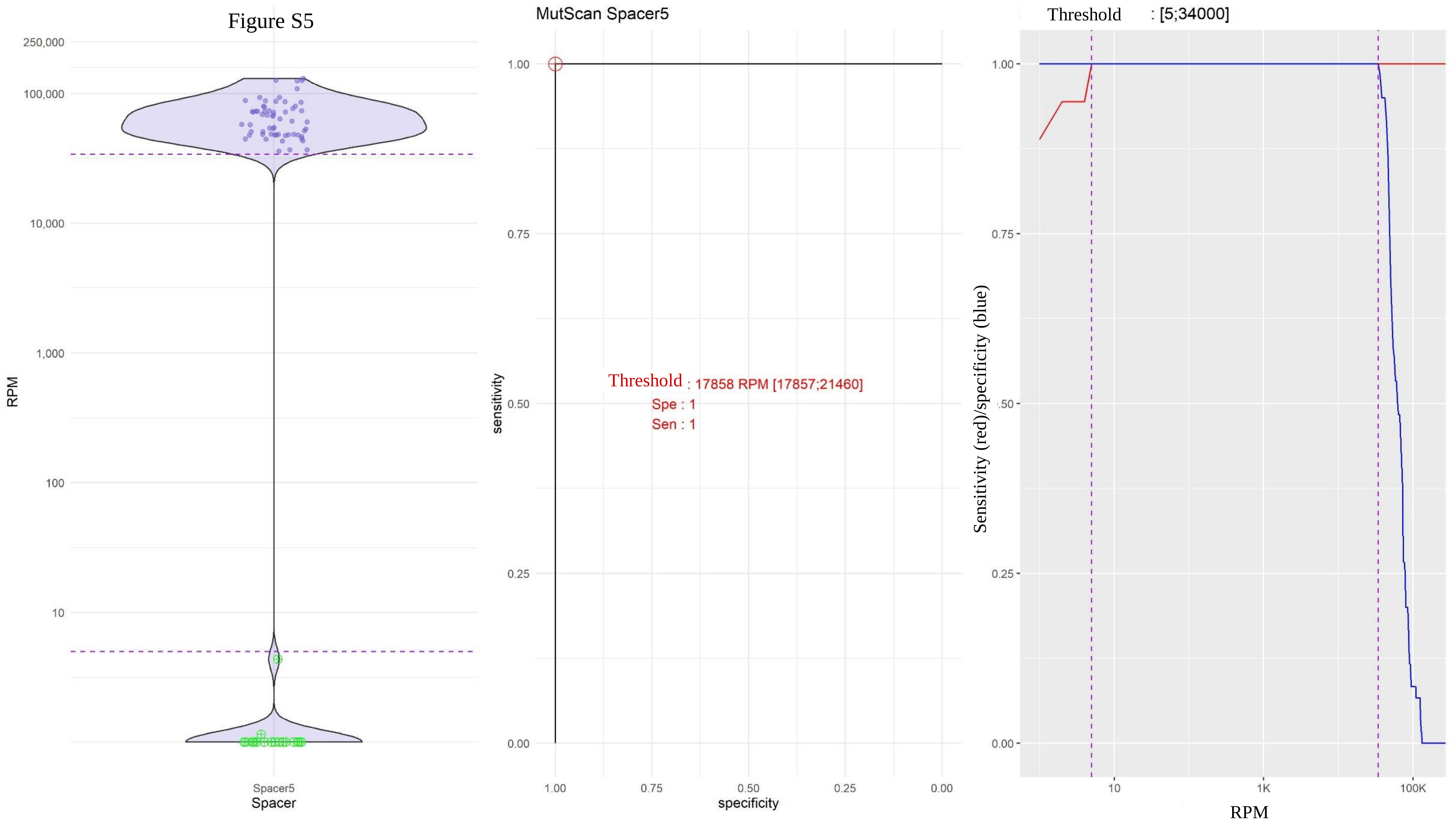
250,000
100,000
10,000
1,000
100
10
RPM
Spacer5
Spacer
MutScan Spacer5
1.00
0.75
0.50
0.25
0.00
sensitivity
specificity
Threshold : 17858 RPM [17857;21460]
Spe : 1
Sen : 1
Threshold : [5;34000]
Sensitivity (red)/specificity (blue)
10
1K
100K
RPM

Figure S6

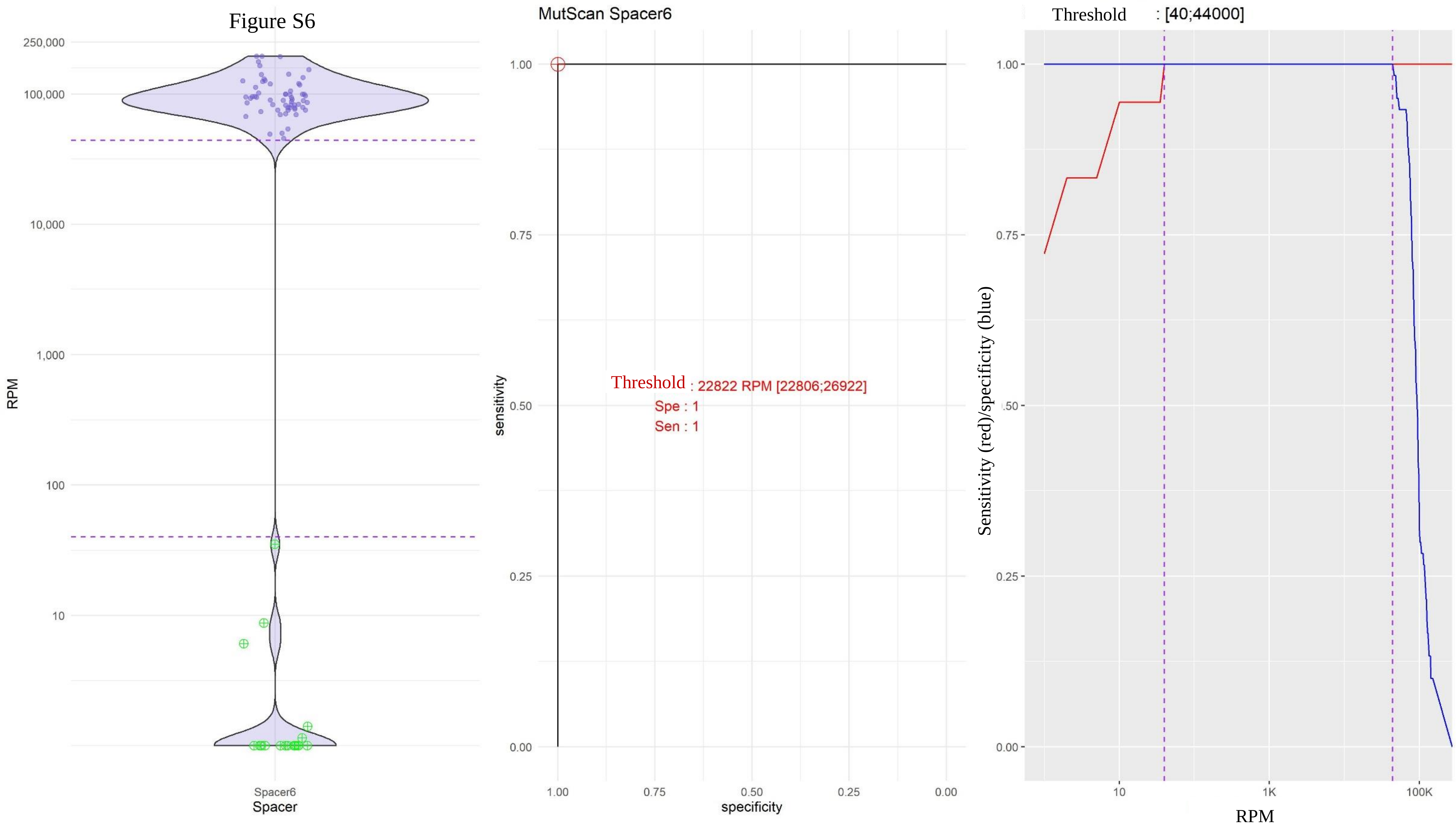

250,000
100,000
10,000
1,000
100
10
RPM
Spacer6
Spacer
MutScan Spacer6
1.00
0.75
0.50
0.25
0.00
sensitivity
specificity
Threshold : 22822 RPM [22806;26922]
Spe : 1
Sen : 1
Threshold : [40;44000]
Sensitivity (red)/specificity (blue)
10
1K
100K
RPM

Figure S7

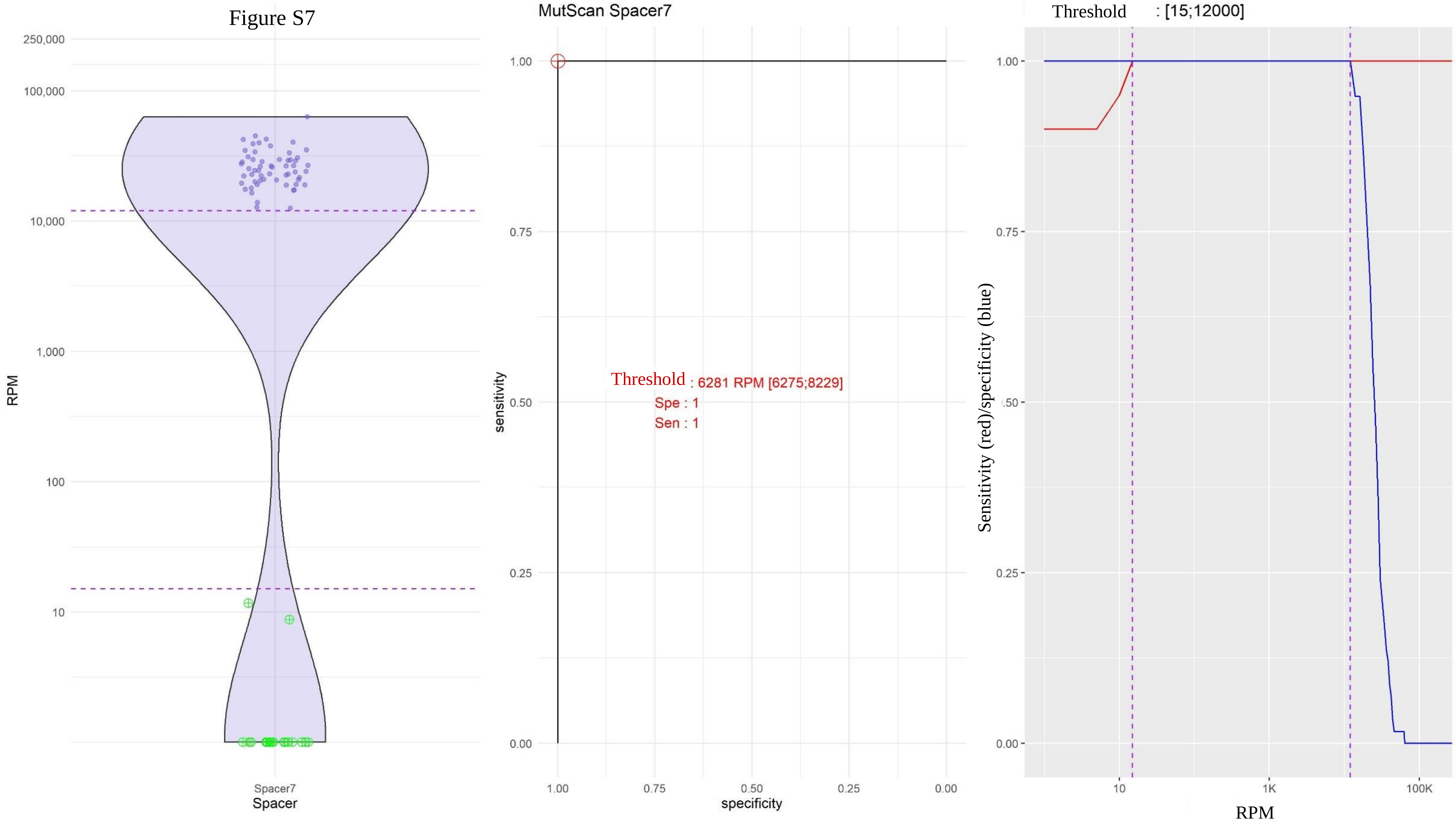
250,000
100,000
10,000
1,000
100
10
RPM
Spacer7
Spacer
MutScan Spacer7
1.00
0.75
0.50
0.25
0.00
sensitivity
specificity
Threshold : 6281 RPM [6275;8229]
Spe : 1
Sen : 1
Threshold : [15;12000]
Sensitivity (red)/specificity (blue)
10
1K
100K
RPM

Figure S8

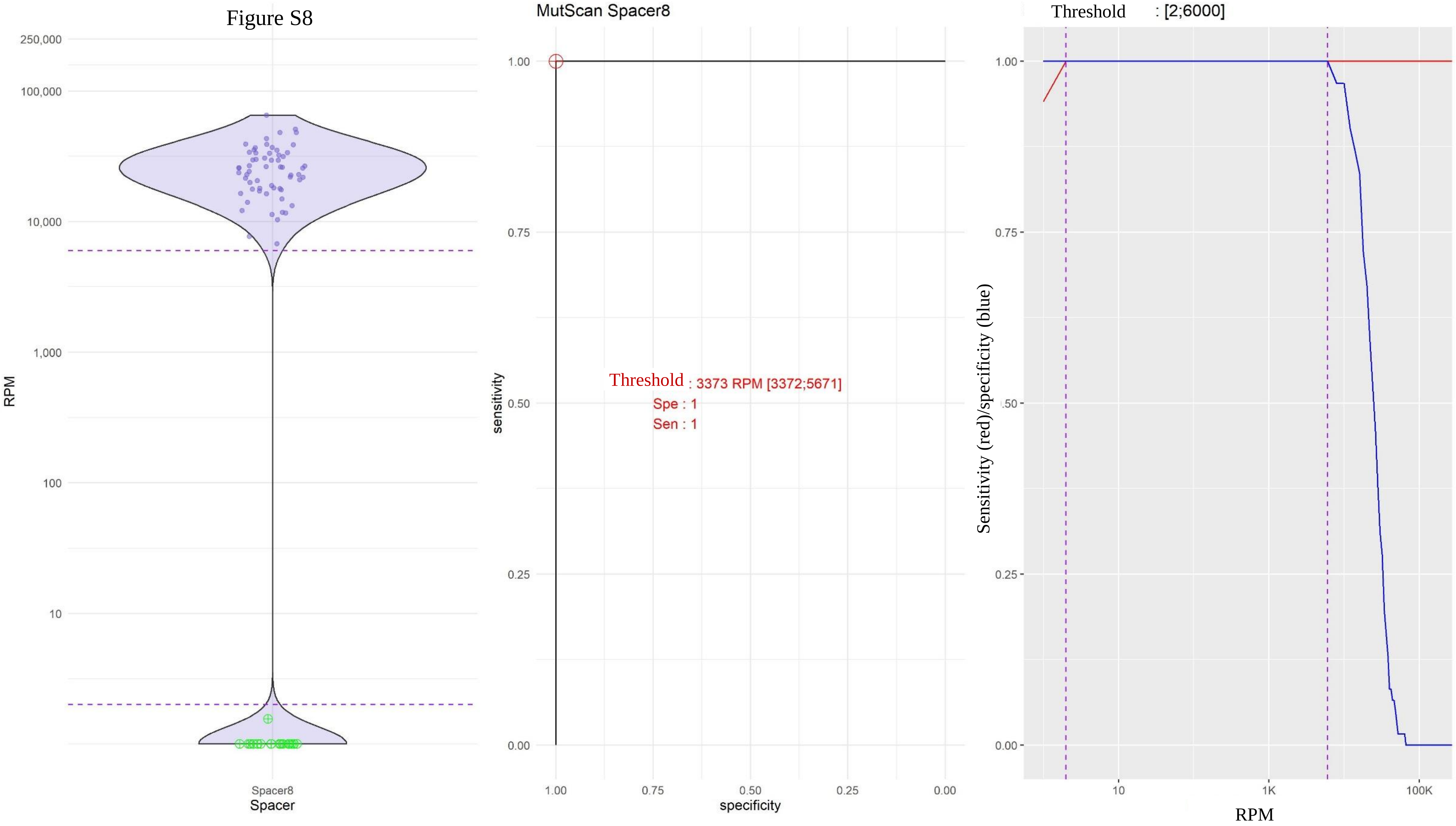
250,000
100,000
10,000
1,000
100
10
RPM
Spacer8
Spacer
MutScan Spacer8
1.00
0.75
0.50
0.25
0.00
sensitivity
specificity
Threshold : 3373 RPM [3372;5671]
Spe : 1
Sen : 1
Threshold : [2;6000]
Sensitivity (red)/specificity (blue)
10
1K
100K
RPM

Figure S9

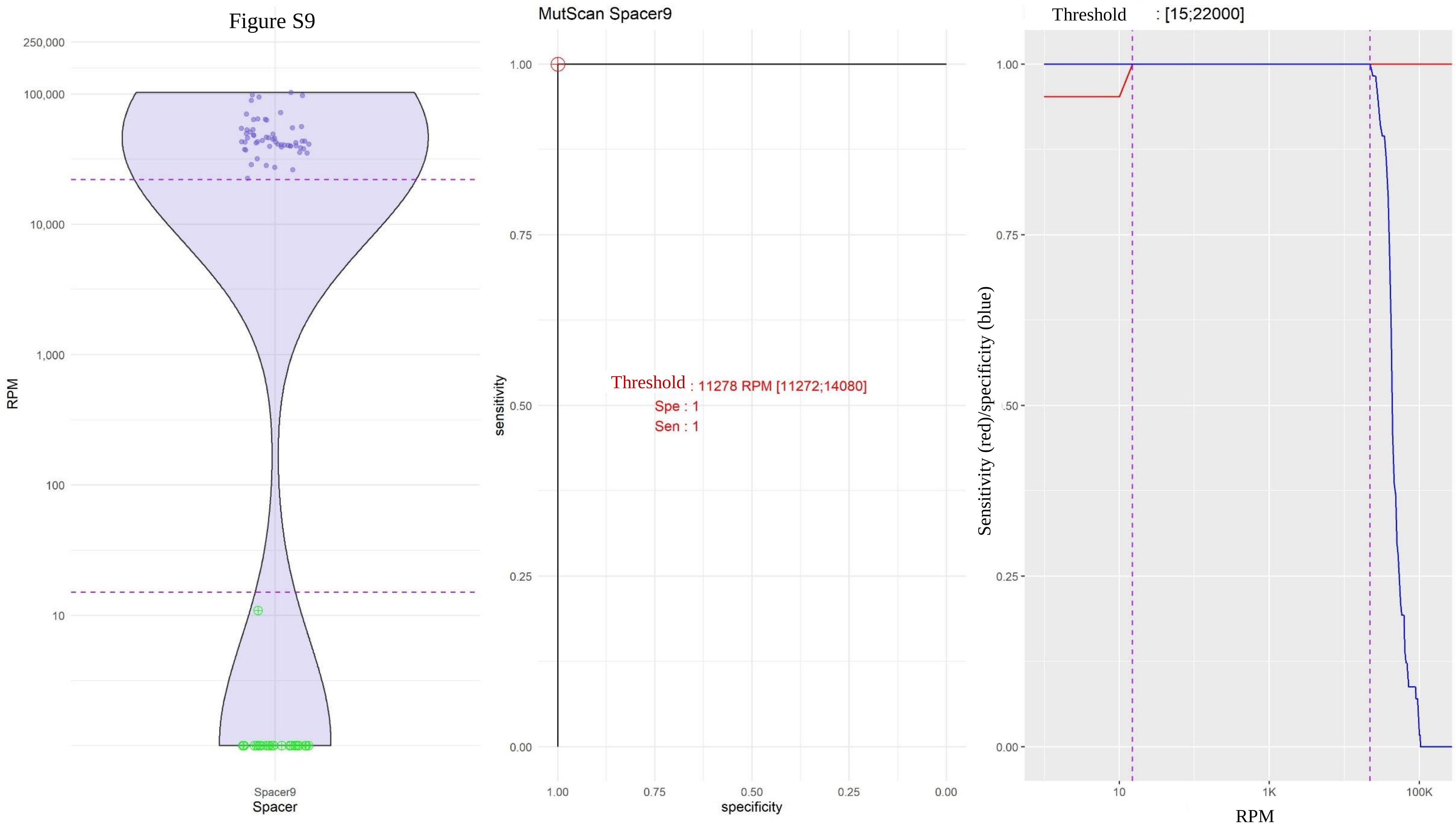
MutScan Spacer9
Threshold : [15;22000]
250,000
100,000
10,000
1,000
100
10
RPM
Spacer9
Spacer
1.00
0.75
0.50
0.25
0.00
sensitivity
specificity
Threshold : 11278 RPM [11272;14080]
Spe : 1
Sen : 1
Sensitivity (red)/specificity (blue)
10
1K
100K
RPM

Figure S10

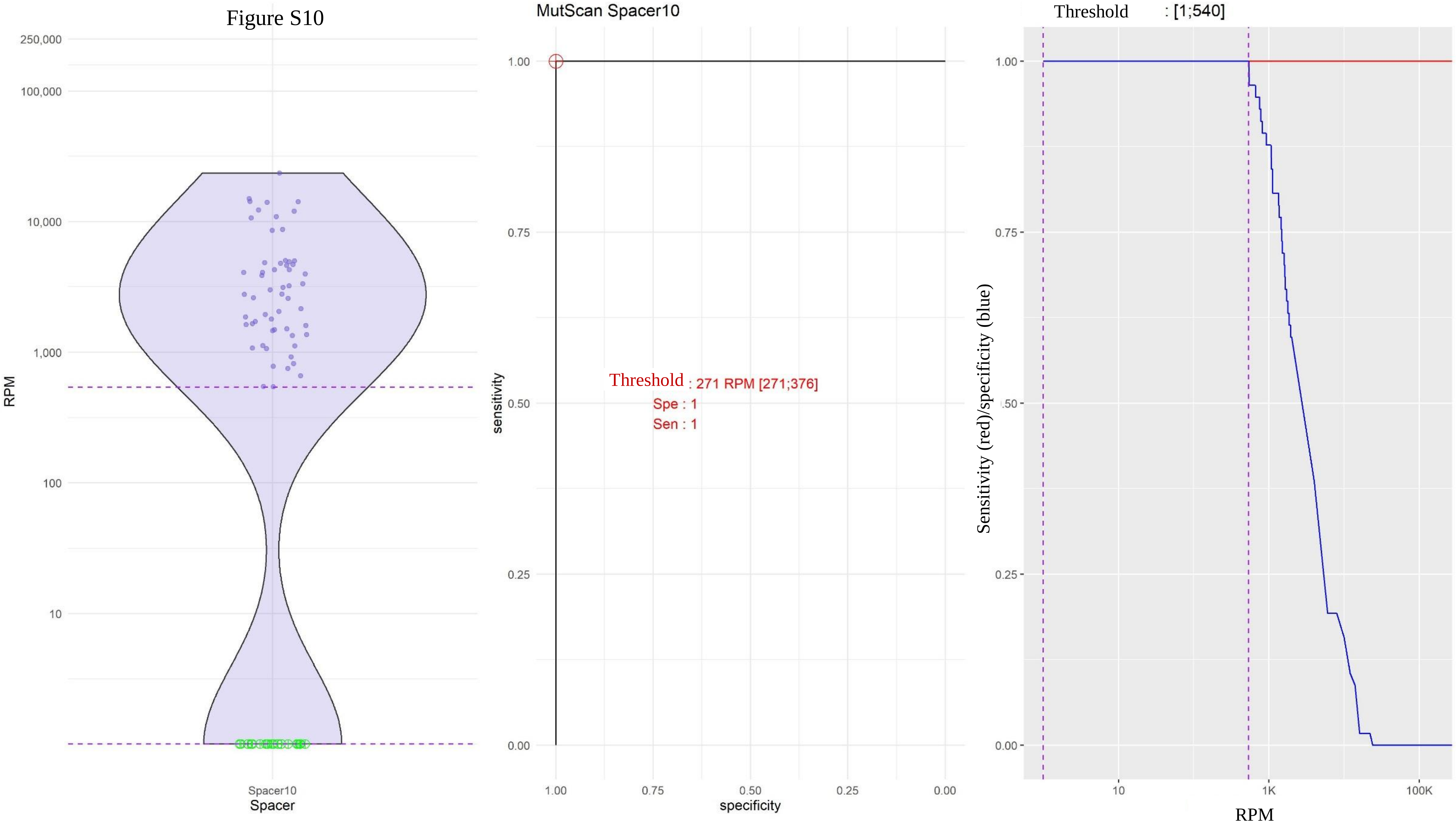

250,000
100,000
10,000
1,000
100
10
RPM
Spacer10
Spacer
MutScan Spacer10
1.00
0.75
0.50
0.25
0.00
sensitivity
specificity
Threshold : 271 RPM [271;376]
Spe : 1
Sen : 1
Threshold : [1;540]
Sensitivity (red)/specificity (blue)
10
1K
100K
RPM

Figure S11

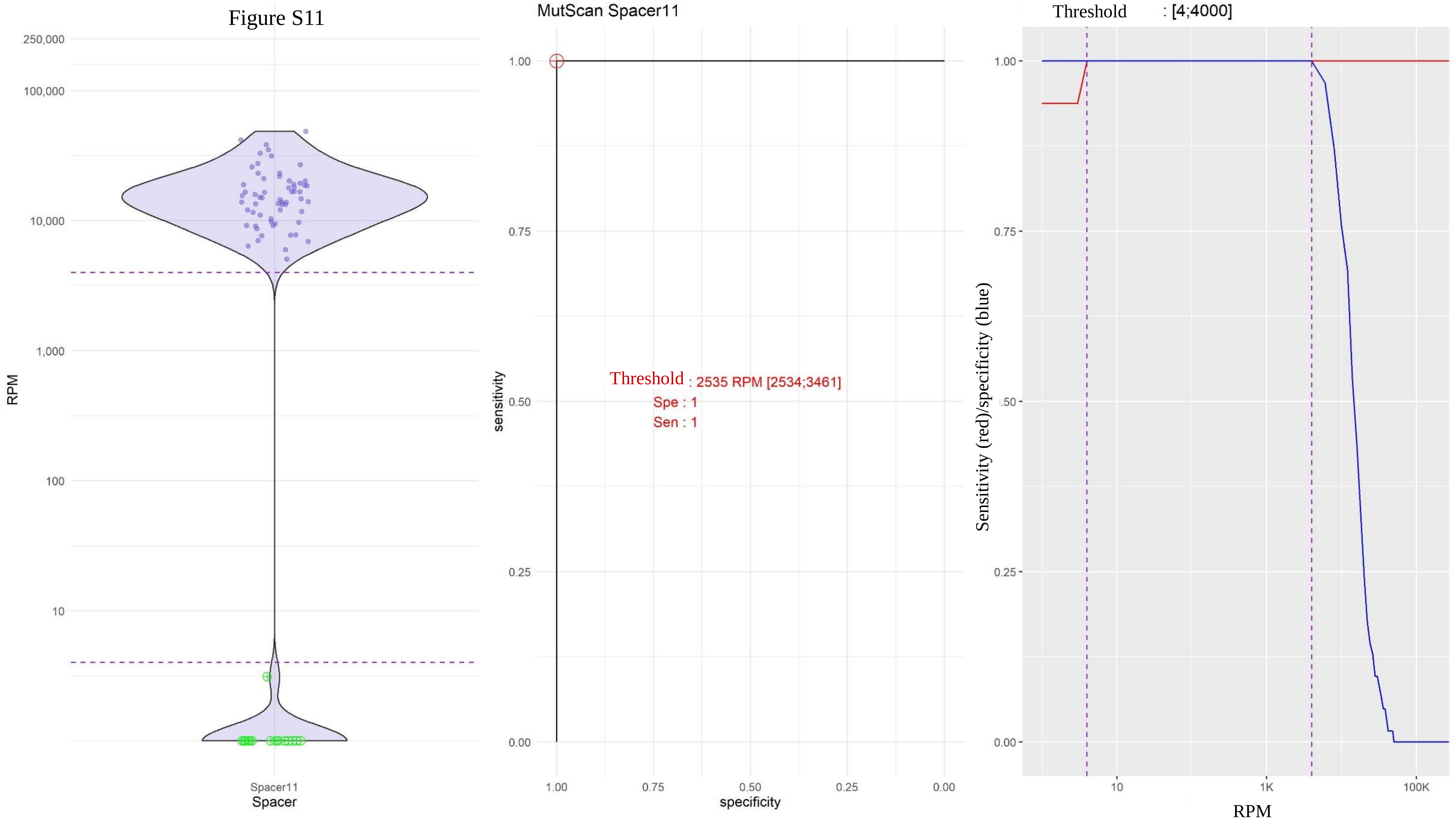

250,000
100,000
10,000
1,000
100
10
RPM
Spacer11
Spacer
MutScan Spacer11
1.00
0.75
0.50
0.25
0.00
sensitivity
specificity
Threshold : 2535 RPM [2534;3461]
Spe : 1
Sen : 1
Threshold : [4;4000]
Sensitivity (red)/specificity (blue)
10
1K
100K
RPM

Figure S12

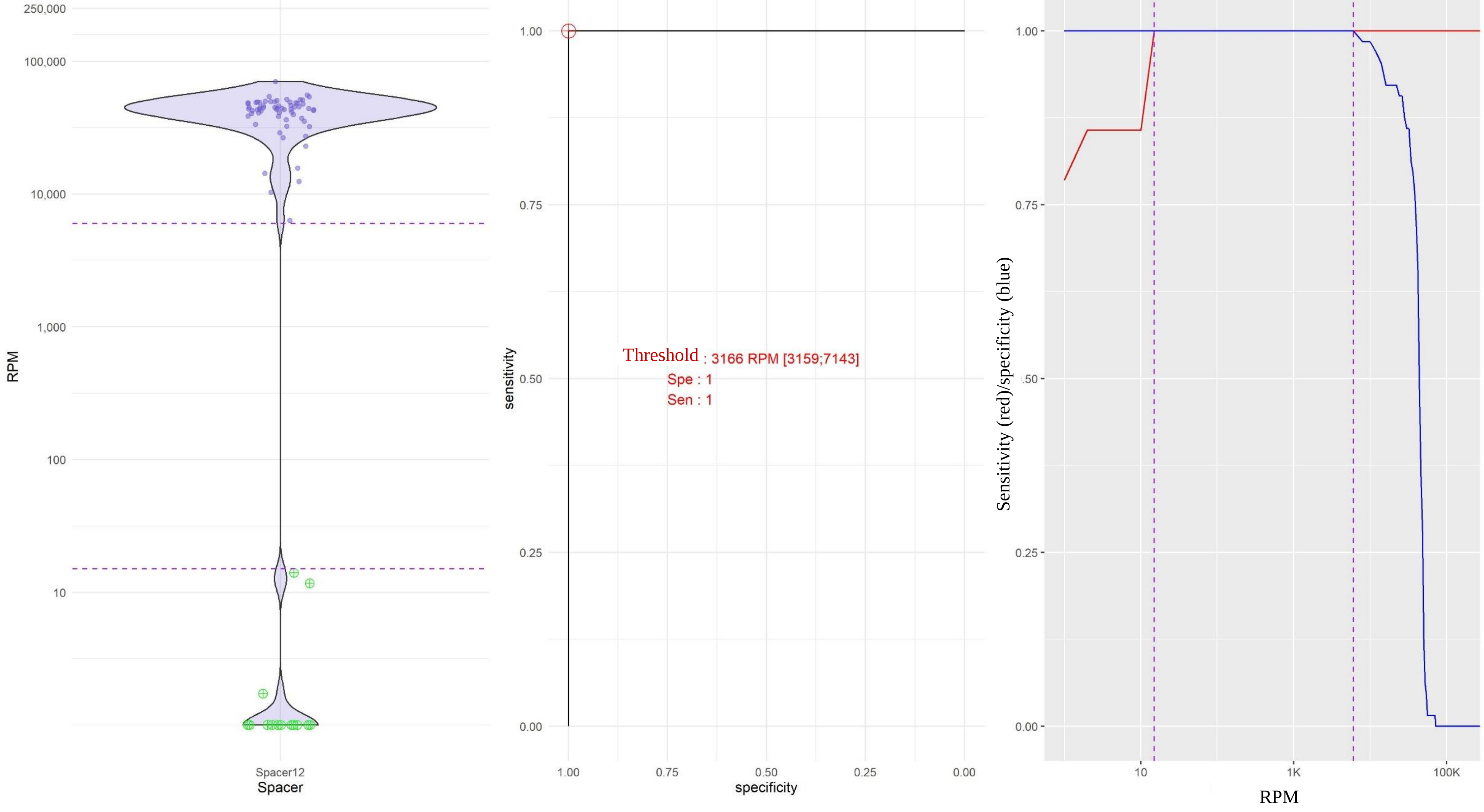
MutScan Spacer12
Threshold : [15;6000]
250,000
100,000
10,000
1,000
100
10
RPM
Spacer12
Spacer
1.00
0.75
0.50
0.25
0.00
sensitivity
specificity
Threshold : 3166 RPM [3159;7143]
Spe : 1
Sen : 1
Sensitivity (red)/specificity (blue)
10
1K
100K
RPM

Figure S13

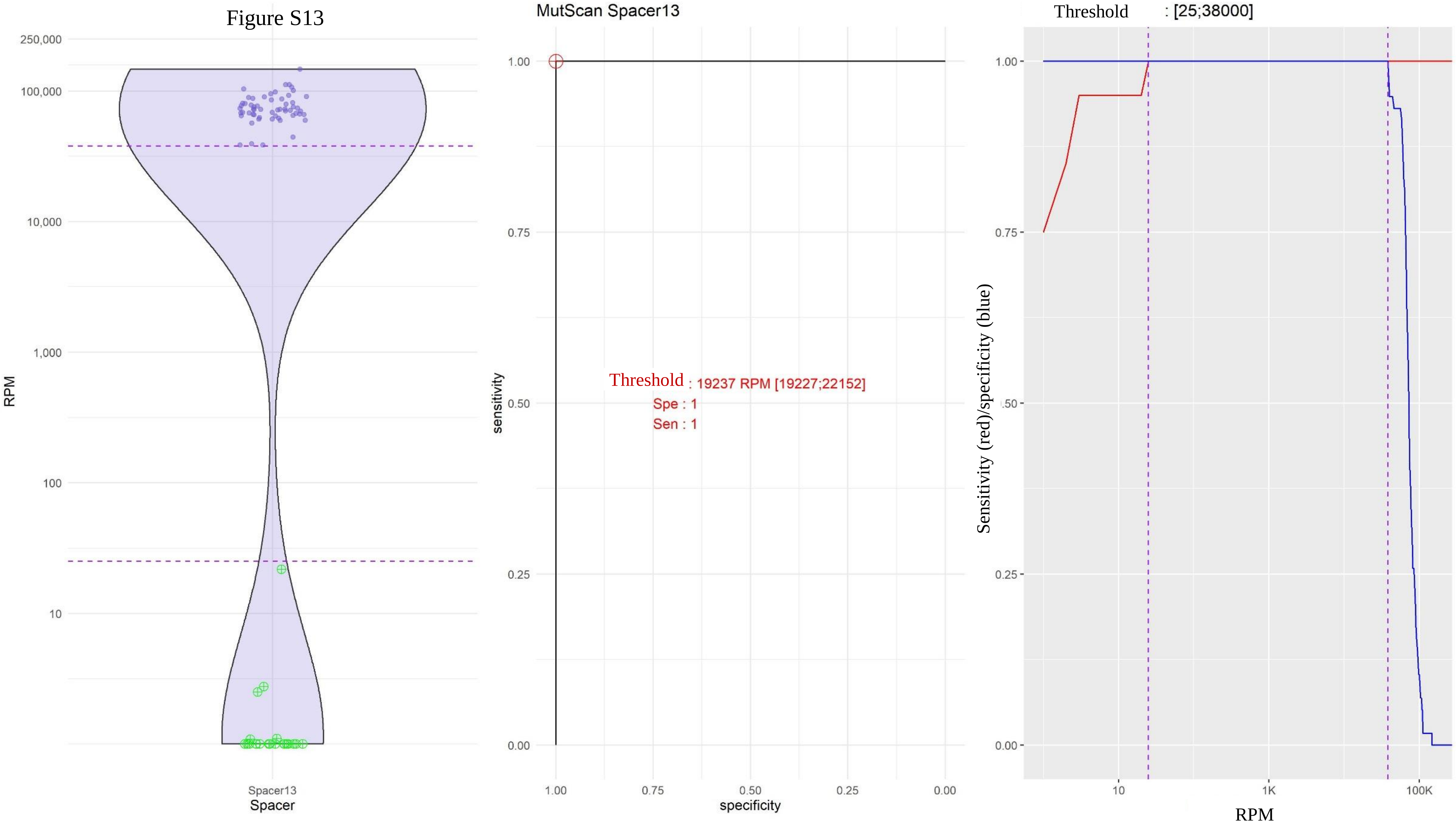
250,000
100,000
10,000
1,000
100
10
RPM
Spacer13
Spacer
MutScan Spacer13
1.00
0.75
0.50
0.25
0.00
sensitivity
Threshold : 19237 RPM [19227;22152]
Spe : 1
Sen : 1
1.00
0.75
0.50
0.25
0.00
specificity
Threshold : [25;38000]
1.00
0.75
.50
0.25
0.00
Sensitivity (red)/specificity (blue)
10
1K
100K
RPM

Figure S14

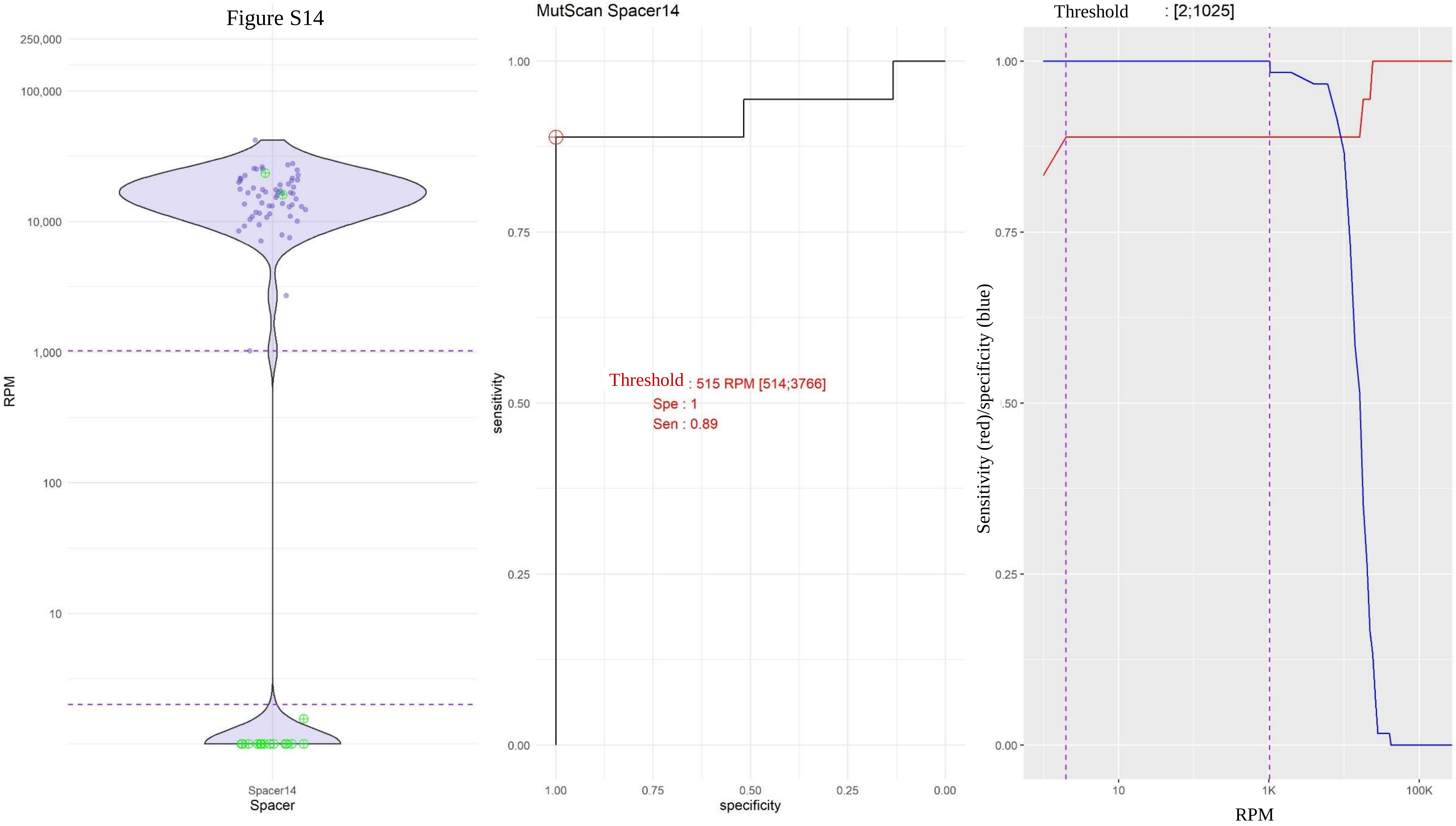
250,000
100,000
10,000
1,000
100
10
RPM
Spacer14
Spacer
MutScan Spacer14
1.00
0.75
0.50
0.25
0.00
sensitivity
specificity
Threshold : 515 RPM [514;3766]
Spe : 1
Sen : 0.89
Threshold : [2;1025]
Sensitivity (red)/specificity (blue)
10
1K
100K
RPM

Figure S15

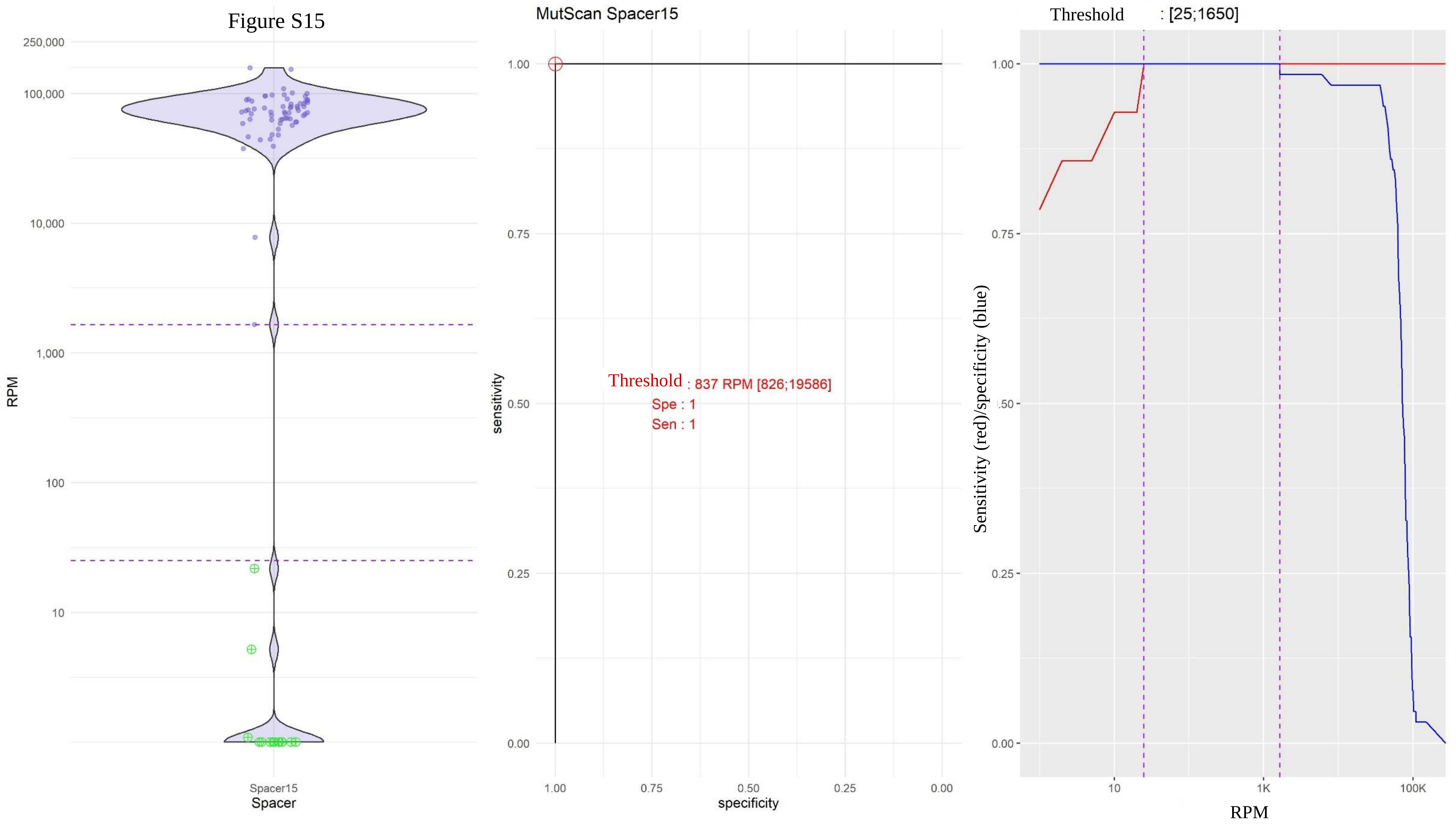

250,000
100,000
10,000
1,000
100
10
RPM
Spacer15
Spacer
MutScan Spacer15
1.00
0.75
0.50
0.25
0.00
sensitivity
specificity
Threshold : 837 RPM [826;19586]
Spe : 1
Sen : 1
Threshold : [25;1650]
Sensitivity (red)/specificity (blue)
10
1K
100K
RPM

Figure S16

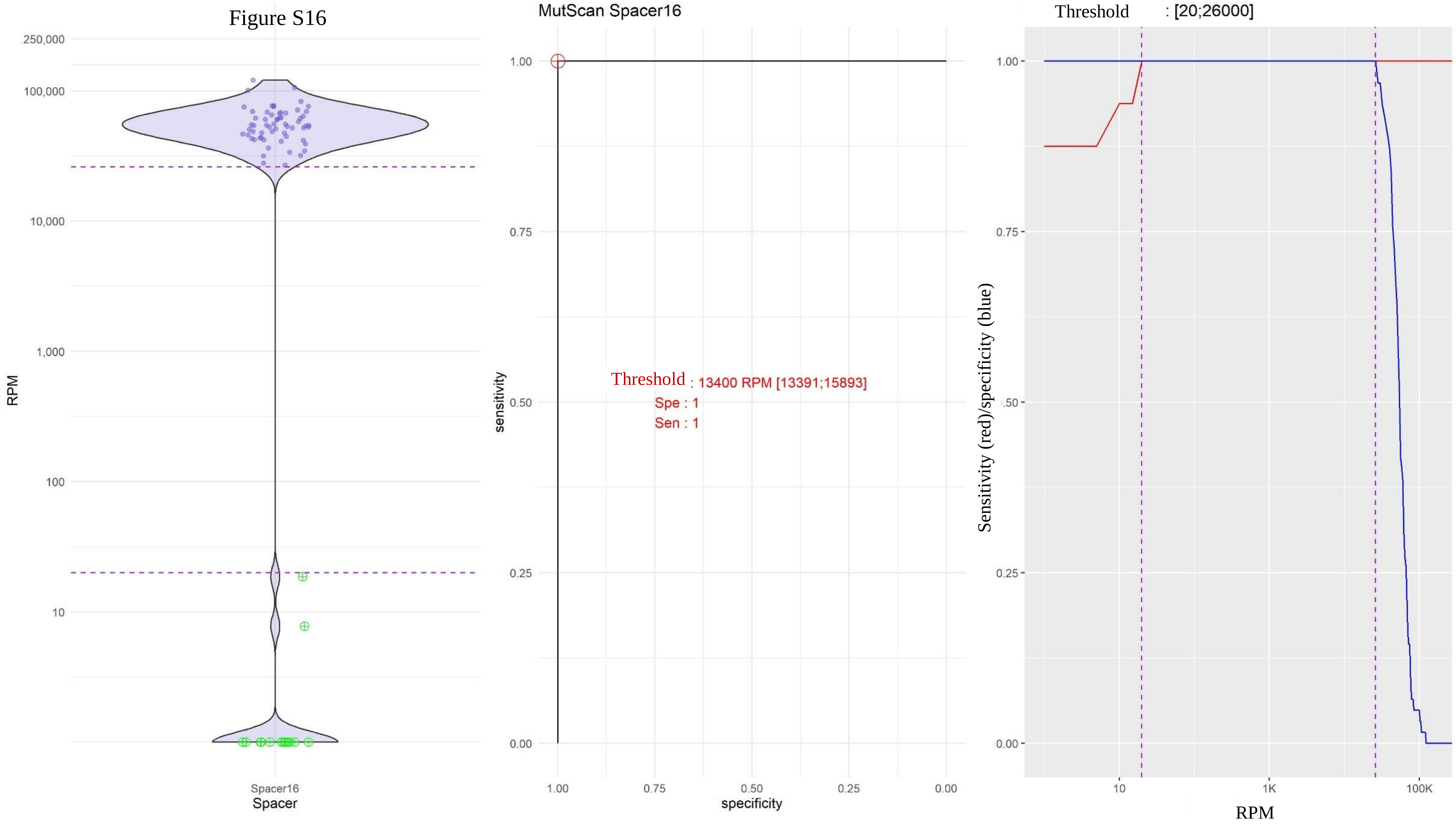
MutScan Spacer16
Threshold : [20;26000]
250,000
100,000
10,000
1,000
100
10
RPM
Spacer16
Spacer
Threshold : 13400 RPM [13391;15893]
Spe : 1
Sen : 1
1.00
0.75
0.50
0.25
0.00
sensitivity
specificity
Sensitivity (red)/specificity (blue)
10
1K
100K
RPM

Figure S17

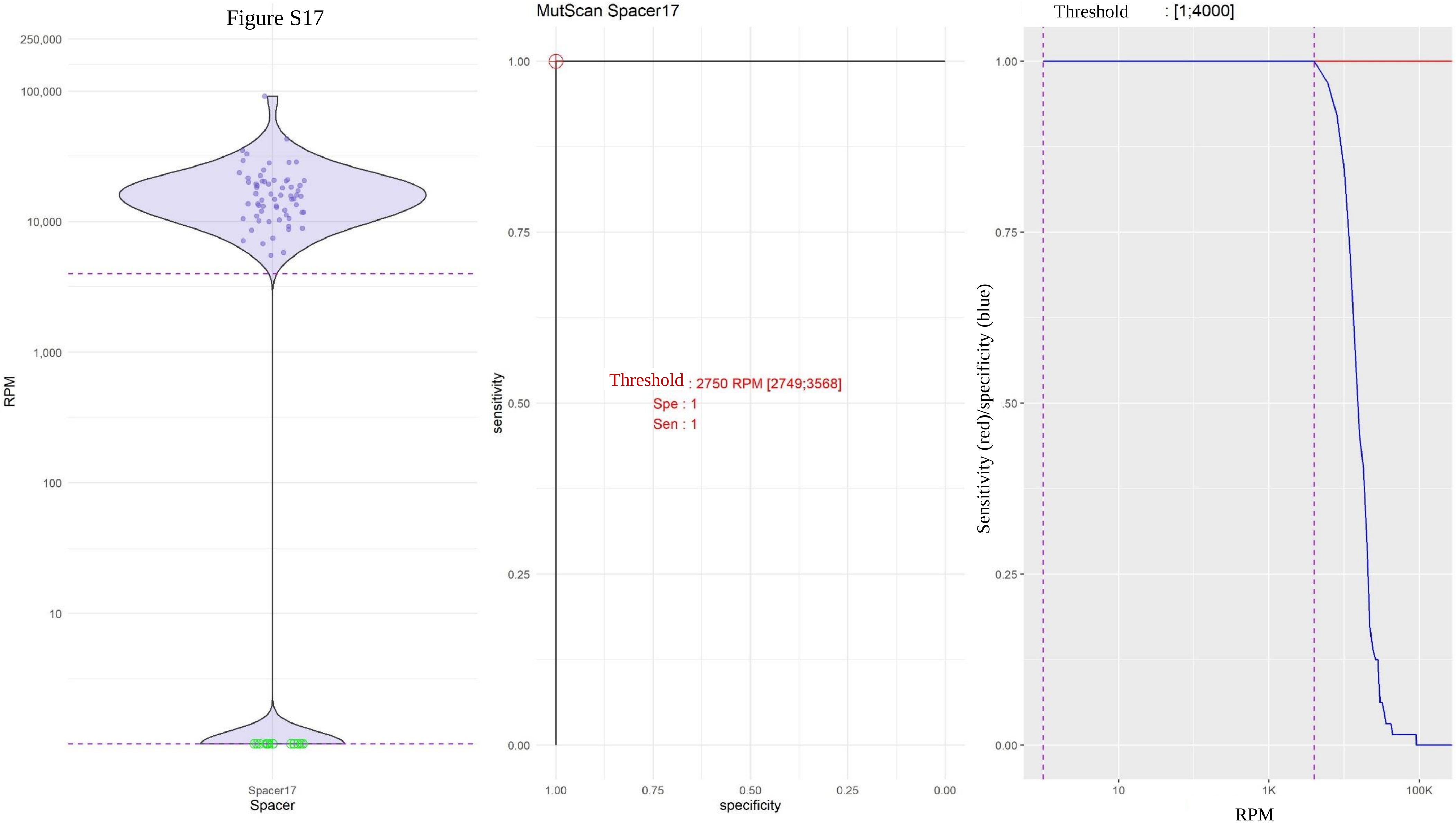
250,000
100,000
10,000
1,000
100
10
RPM
Spacer17
Spacer
MutScan Spacer17
1.00
0.75
0.50
0.25
0.00
sensitivity
specificity
Threshold : 2750 RPM [2749;3568]
Spe : 1
Sen : 1
Threshold : [1;4000]
Sensitivity (red)/specificity (blue)
10
1K
100K
RPM

Figure S18

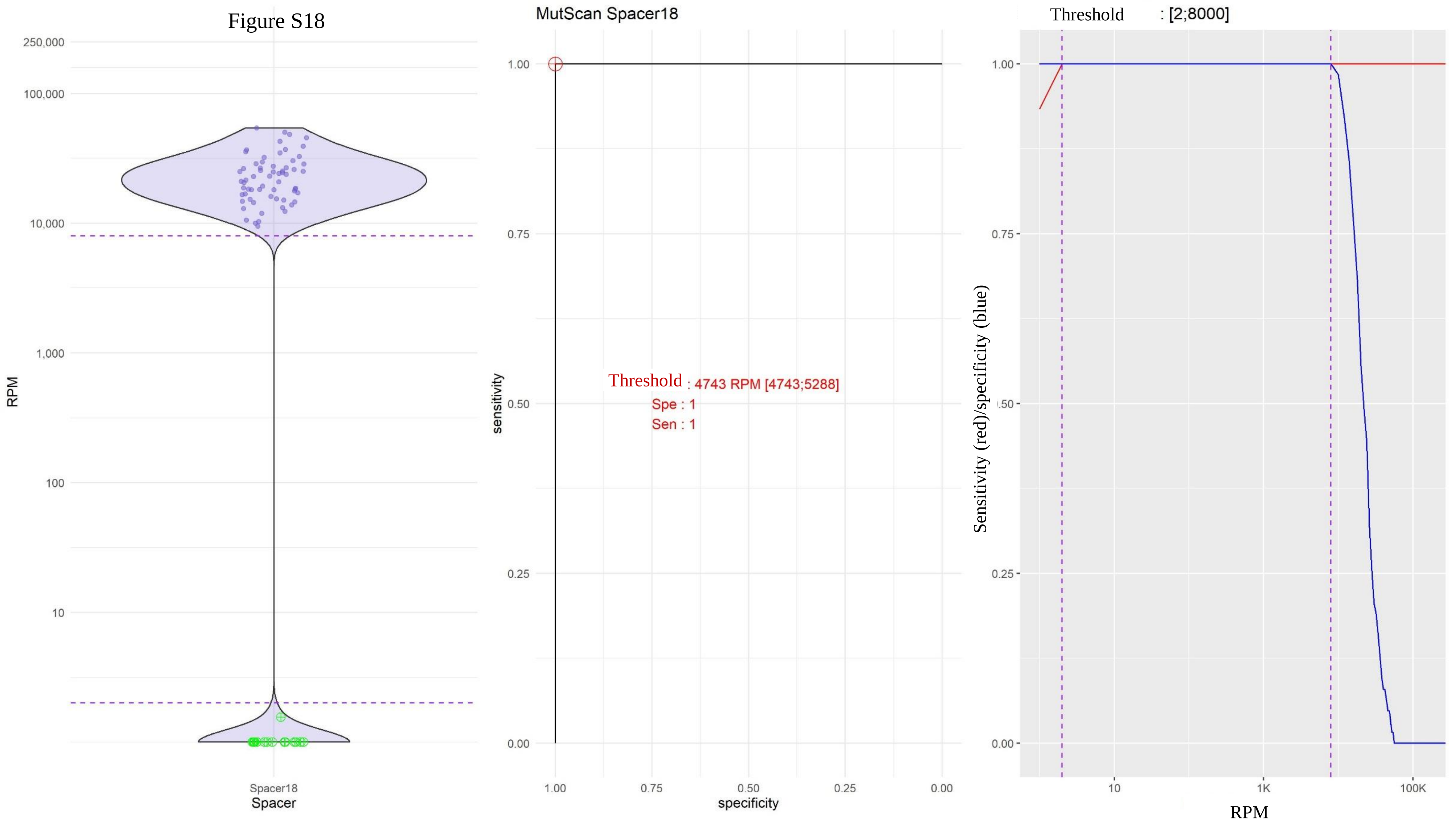
250,000
100,000
10,000
1,000
100
10
RPM
Spacer18
Spacer
MutScan Spacer18
1.00
0.75
0.50
0.25
0.00
sensitivity
specificity
Threshold : 4743 RPM [4743;5288]
Spe : 1
Sen : 1
Threshold : [2;8000]
Sensitivity (red)/specificity (blue)
10
1K
100K
RPM

Figure S19

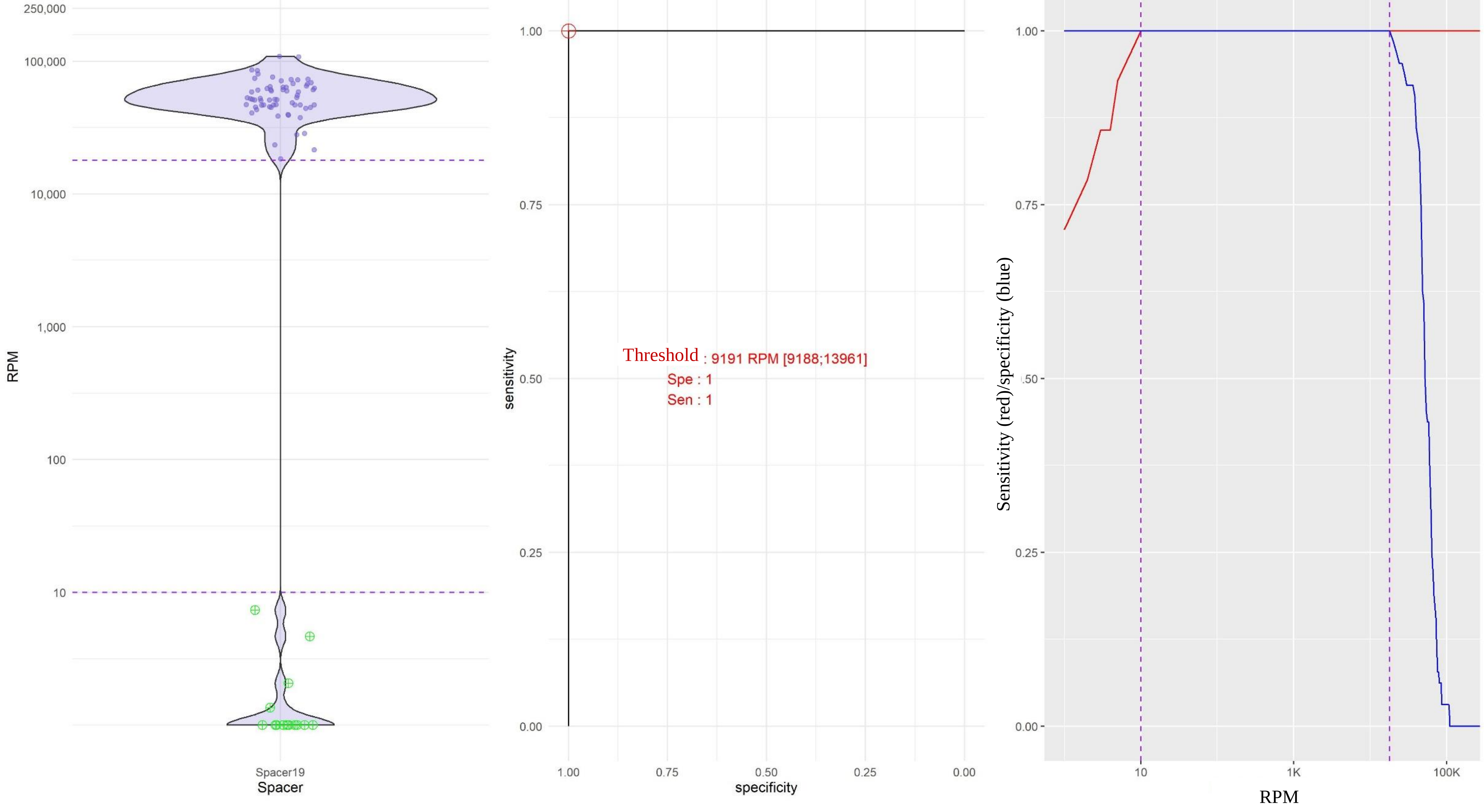

250,000
100,000
10,000
1,000
100
10
RPM
Spacer19
Spacer
MutScan Spacer19
1.00
0.75
0.50
0.25
0.00
sensitivity
specificity
Threshold : 9191 RPM [9188;13961]
Spe : 1
Sen : 1
Threshold : [10;18000]
Sensitivity (red)/specificity (blue)
10
1K
100K
RPM

Figure S20

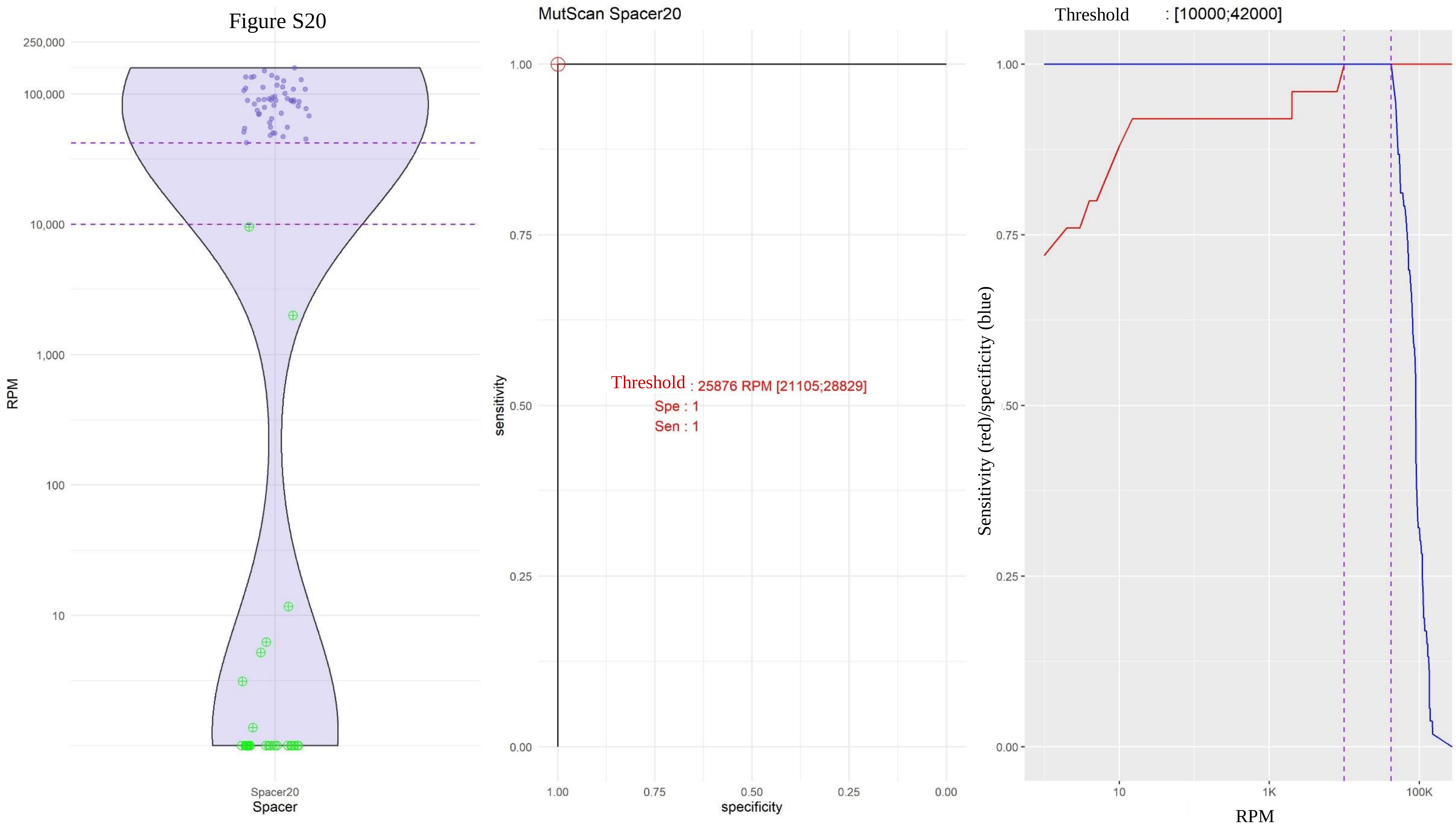

250,000
100,000
10,000
1,000
100
10
RPM
Spacer20
Spacer
MutScan Spacer20
1.00
0.75
0.50
0.25
0.00
sensitivity
specificity
Threshold : 25876 RPM [21105;28829]
Spe : 1
Sen : 1
Threshold : [10000;42000]
Sensitivity (red)/specificity (blue)
10
1K
100K
RPM

Figure S21

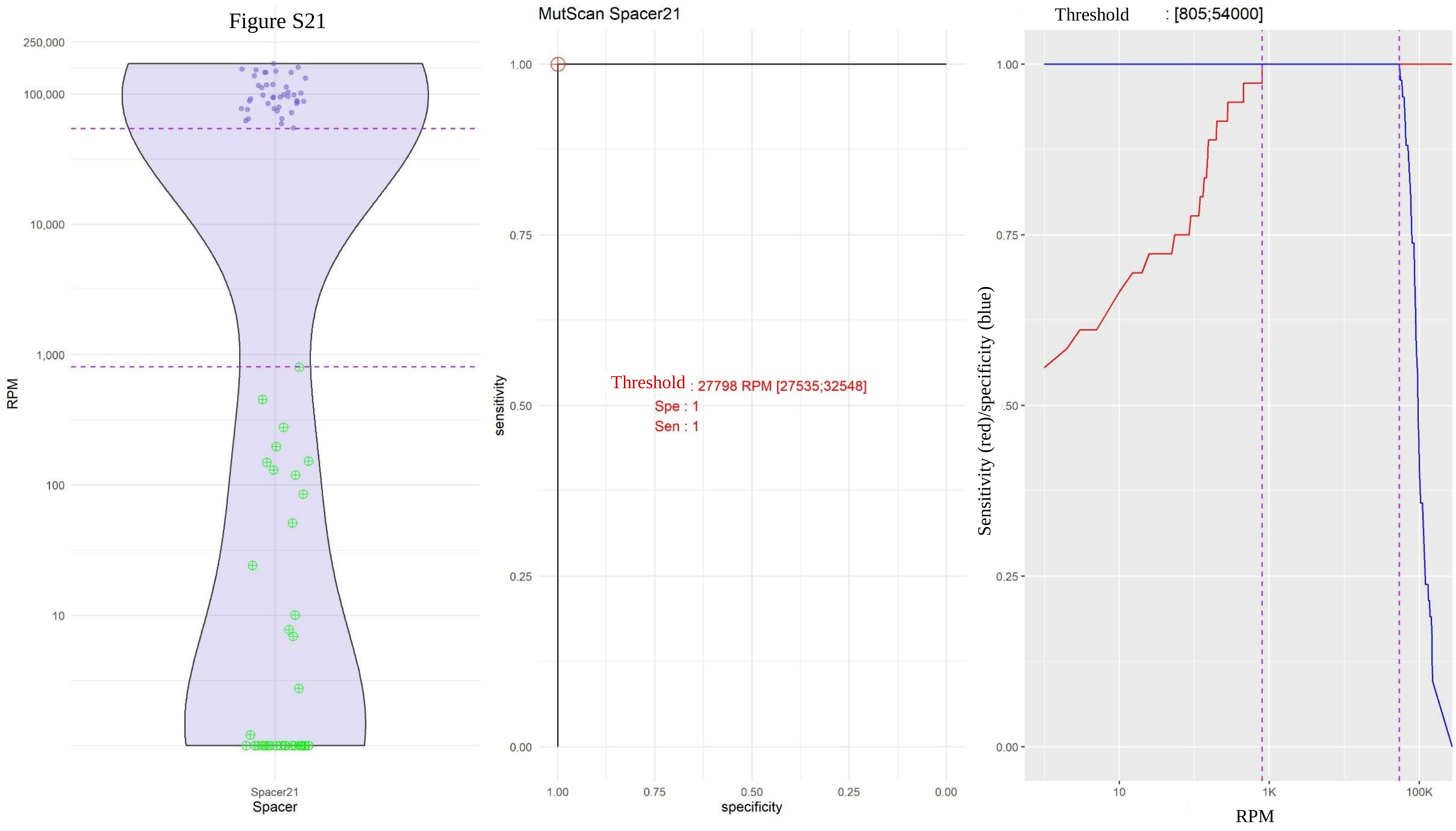

MutScan Spacer21
Threshold : [805;54000]
250,000
100,000
10,000
1,000
100
10
RPM
Spacer21
Spacer
1.00
0.75
0.50
0.25
0.00
sensitivity
specificity
Threshold : 27798 RPM [27535;32548]
Spe : 1
Sen : 1
Sensitivity (red)/specificity (blue)
10
1K
100K
RPM

Figure S22

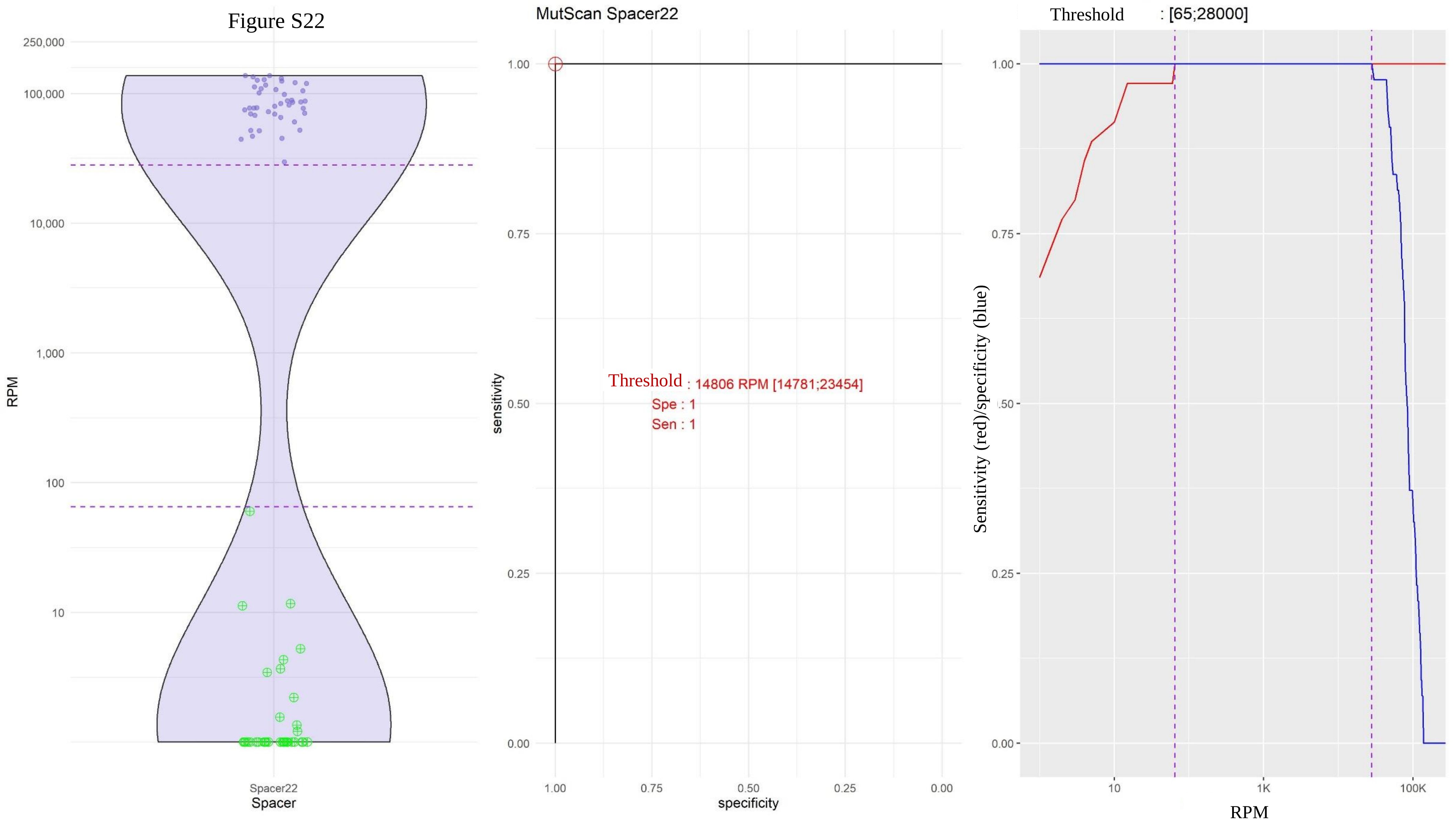
250,000
100,000
10,000
1,000
100
10
RPM
Spacer22
Spacer
MutScan Spacer22
1.00
0.75
0.50
0.25
0.00
sensitivity
specificity
Threshold : 14806 RPM [14781;23454]
Spe : 1
Sen : 1
Threshold : [65;28000]
Sensitivity (red)/specificity (blue)
10
1K
100K
RPM

Figure S23

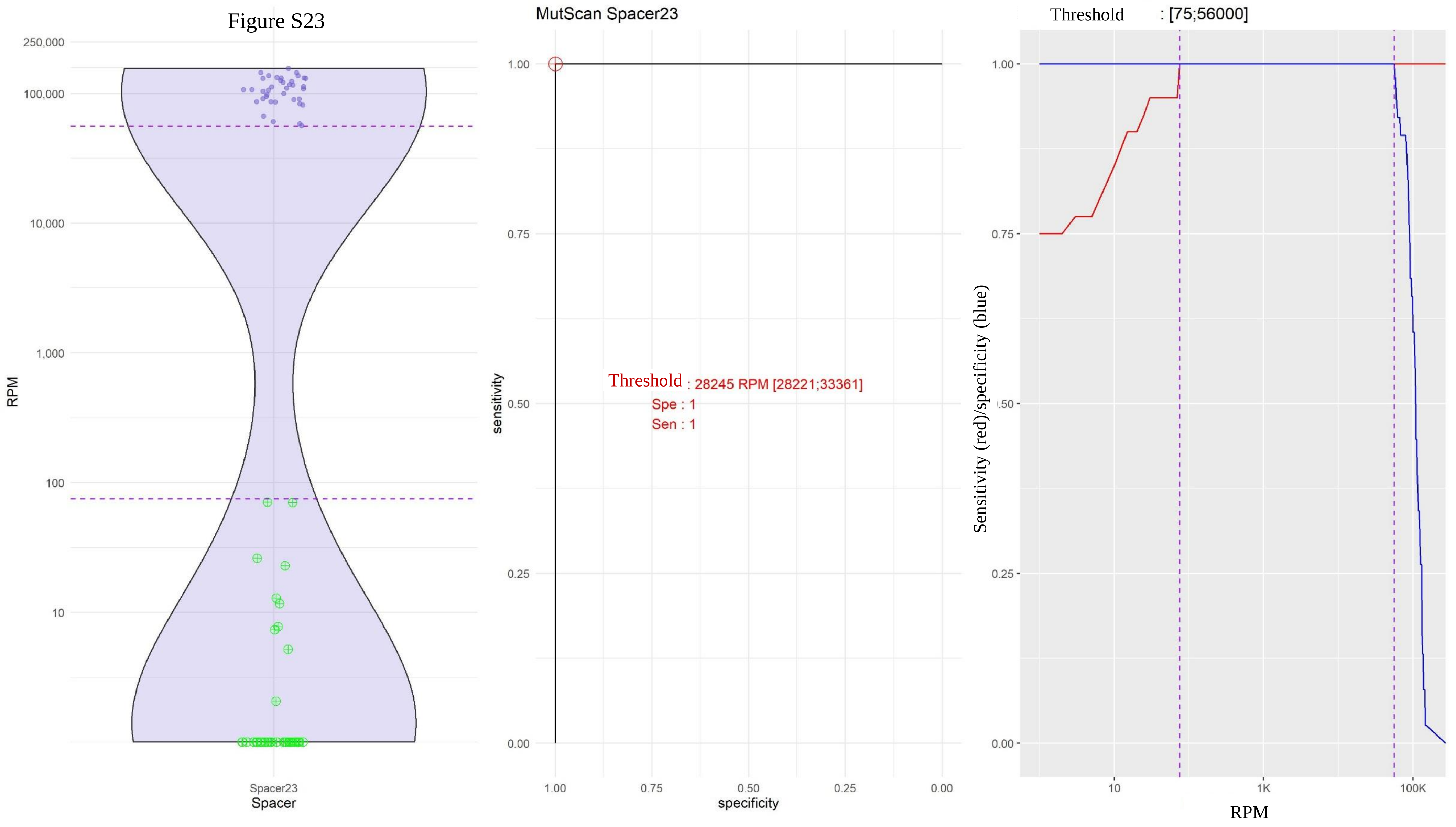
250,000
100,000
10,000
1,000
100
10
RPM
Spacer23
Spacer
MutScan Spacer23
1.00
0.75
0.50
0.25
0.00
sensitivity
specificity
Threshold : 28245 RPM [28221;33361]
Spe : 1
Sen : 1
Threshold : [75;56000]
Sensitivity (red)/specificity (blue)
10
1K
100K
RPM

Figure S24

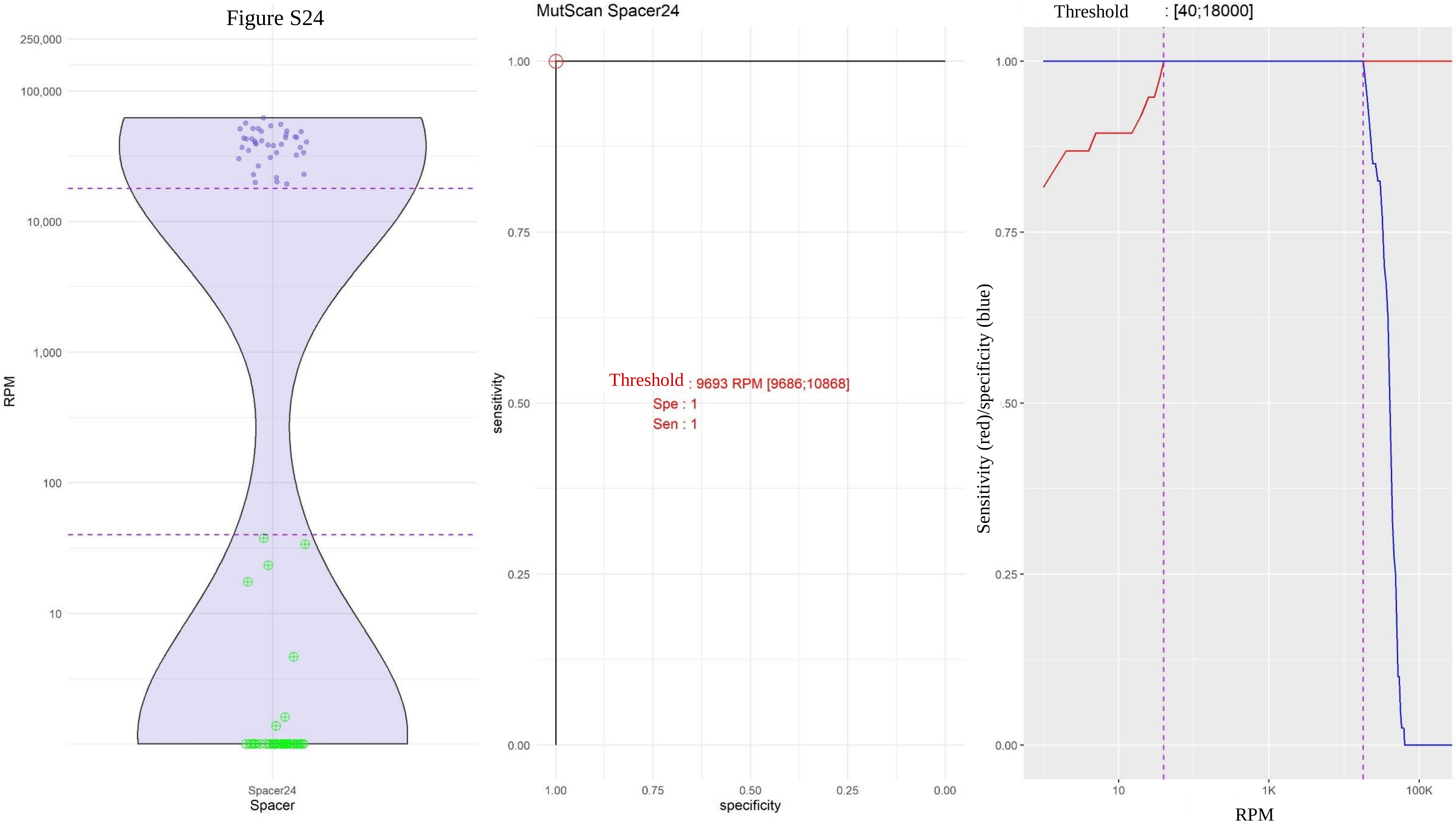
MutScan Spacer24
Threshold : [40;18000]
Threshold : 9693 RPM [9686;10868]
Spe : 1
Sen : 1
RPM
250,000
100,000
10,000
1,000
100
10
Spacer24
Spacer
sensitivity
1.00
0.75
0.50
0.25
0.00
specificity
Sensitivity (red)/specificity (blue)
10
1K
100K
RPM

Figure S25

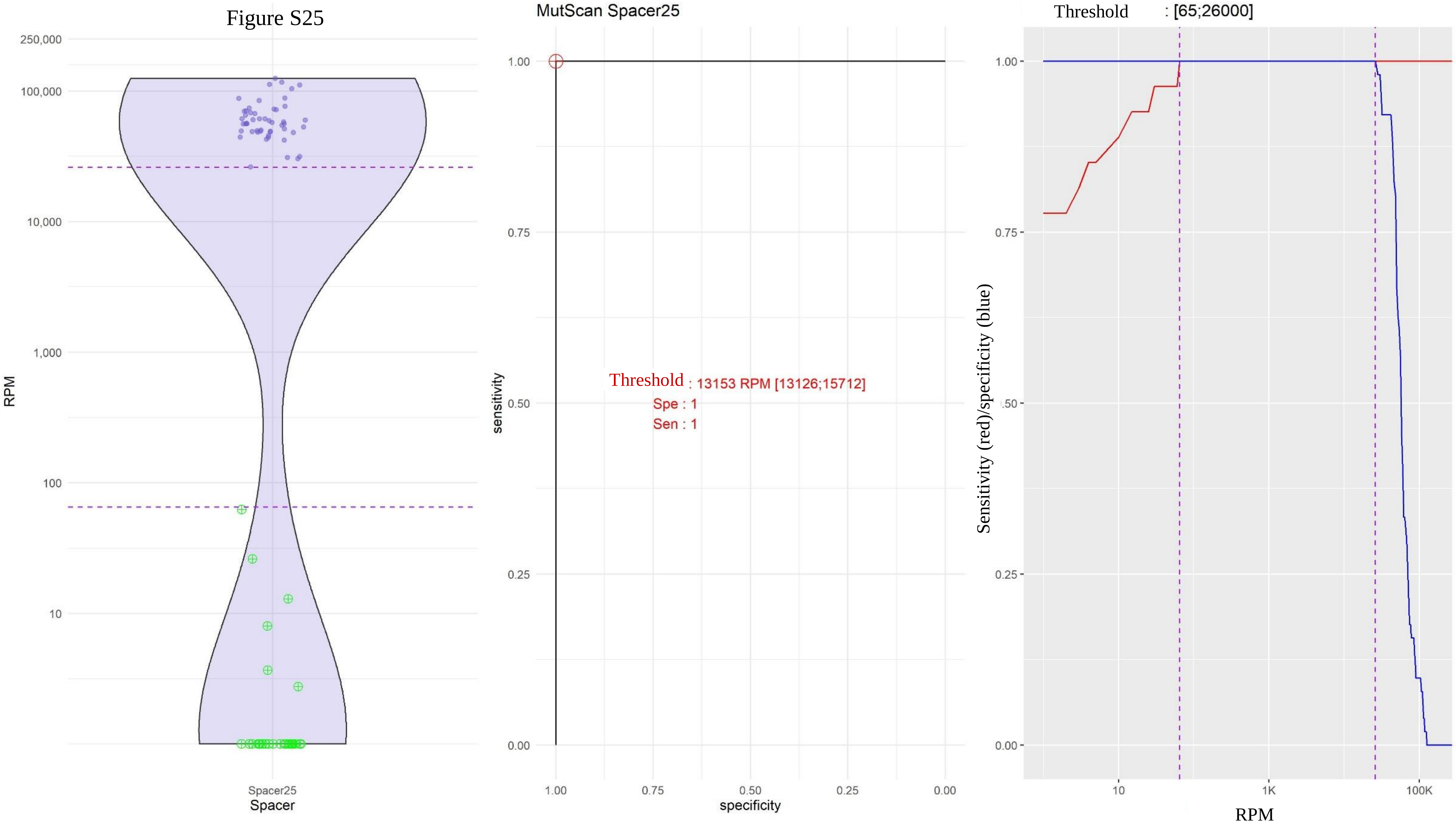

250,000
100,000
10,000
1,000
100
10
RPM
Spacer25
Spacer
MutScan Spacer25
1.00
0.75
0.50
0.25
0.00
sensitivity
specificity
Threshold : 13153 RPM [13126;15712]
Spe : 1
Sen : 1
Threshold : [65;26000]
Sensitivity (red)/specificity (blue)
10
1K
100K
RPM

Figure S26

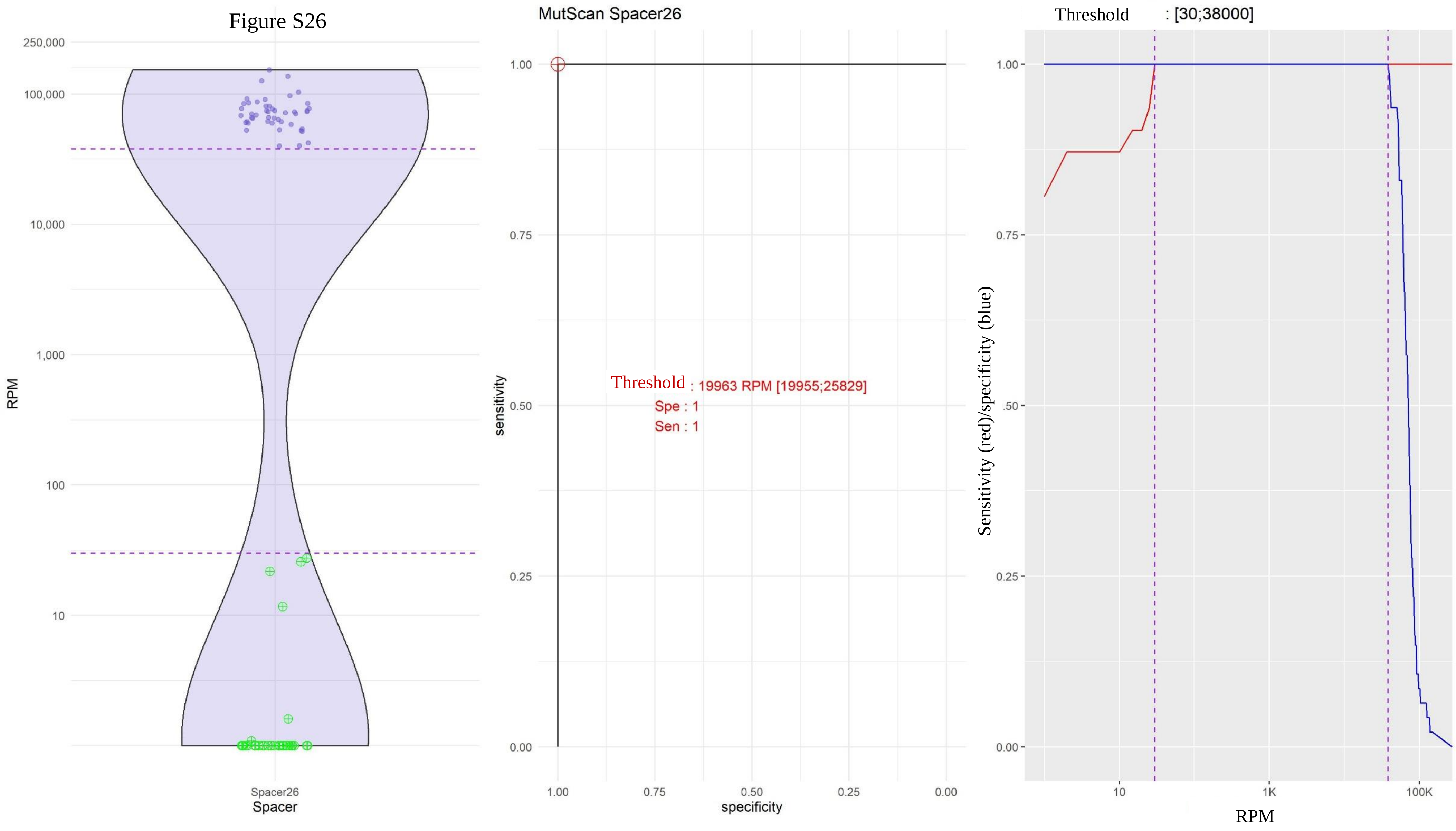
250,000
100,000
10,000
1,000
100
10
RPM
Spacer26
Spacer
MutScan Spacer26
1.00
0.75
0.50
0.25
0.00
sensitivity
specificity
Threshold : 19963 RPM [19955;25829]
Spe : 1
Sen : 1
Threshold : [30;38000]
Sensitivity (red)/specificity (blue)
10
1K
100K
RPM

Figure S27

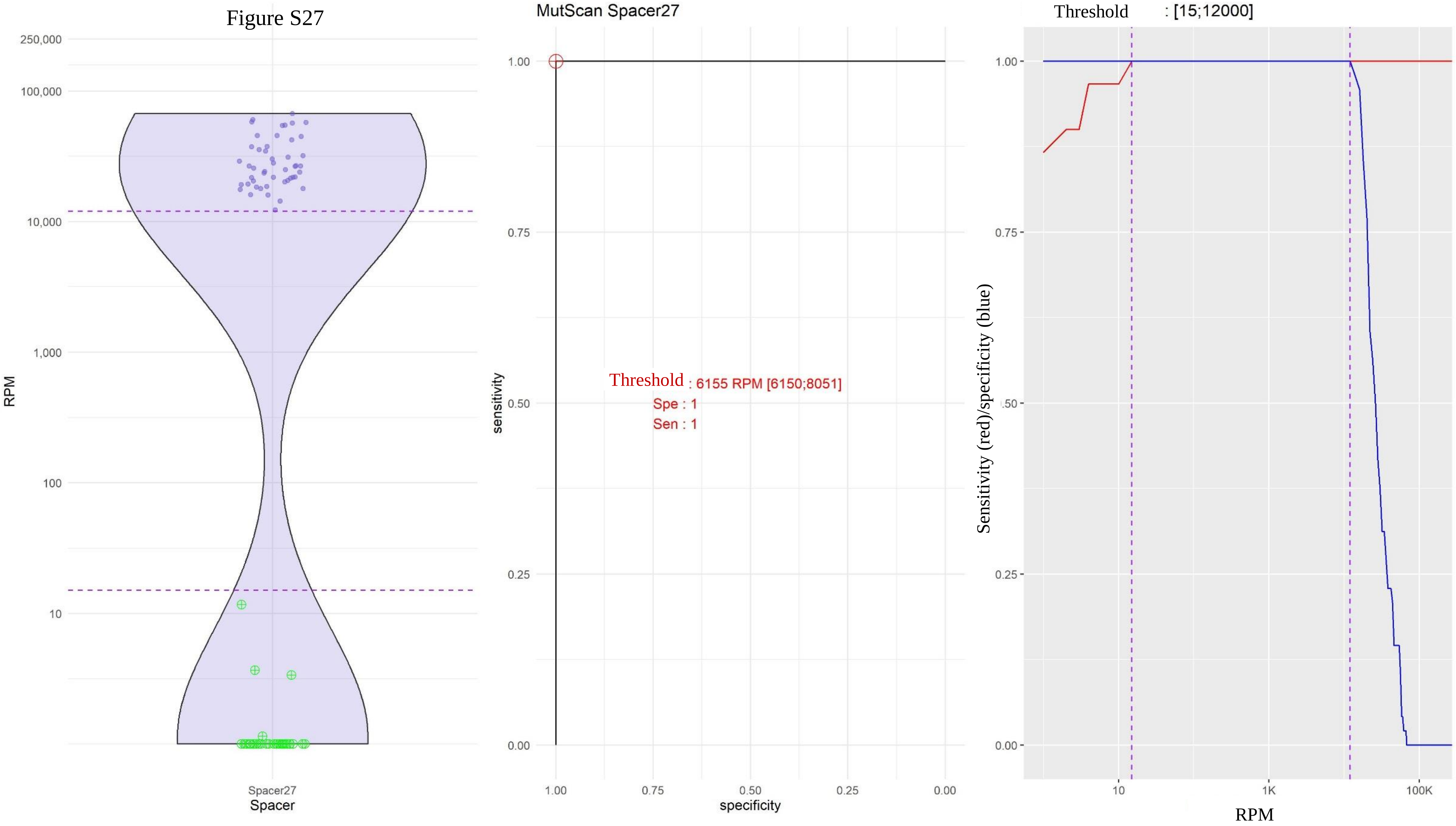
250,000
100,000
10,000
1,000
100
10
RPM
Spacer27
Spacer
MutScan Spacer27
1.00
0.75
0.50
0.25
0.00
sensitivity
specificity
Threshold : 6155 RPM [6150;8051]
Spe : 1
Sen : 1
Threshold : [15;12000]
Sensitivity (red)/specificity (blue)
10
1K
100K
RPM

Figure S28

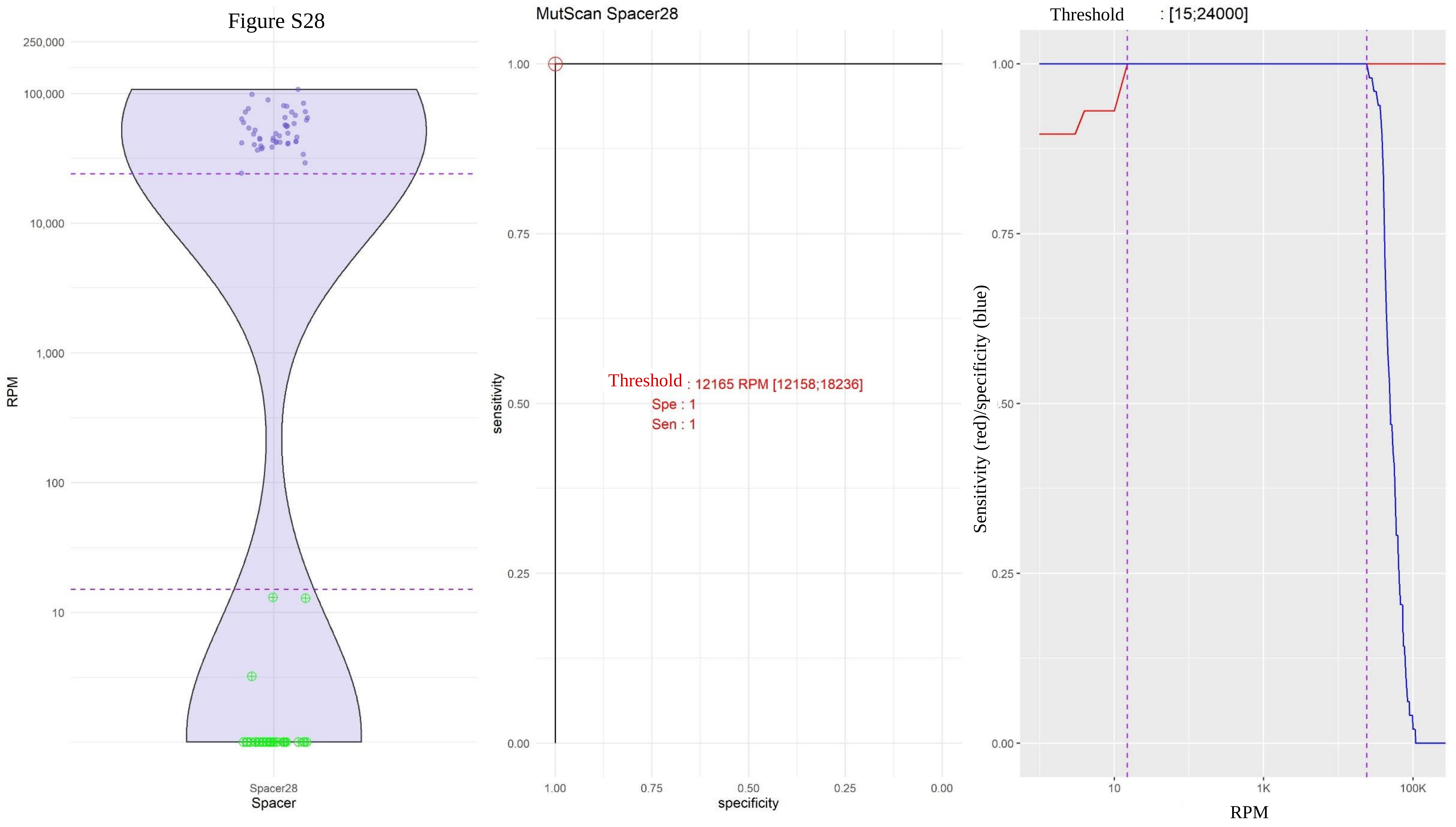
250,000
100,000
10,000
1,000
100
10
RPM
Spacer28
Spacer
MutScan Spacer28
1.00
0.75
0.50
0.25
0.00
sensitivity
specificity
Threshold : 12165 RPM [12158;18236]
Spe : 1
Sen : 1
Threshold : [15;24000]
Sensitivity (red)/specificity (blue)
10
1K
100K
RPM

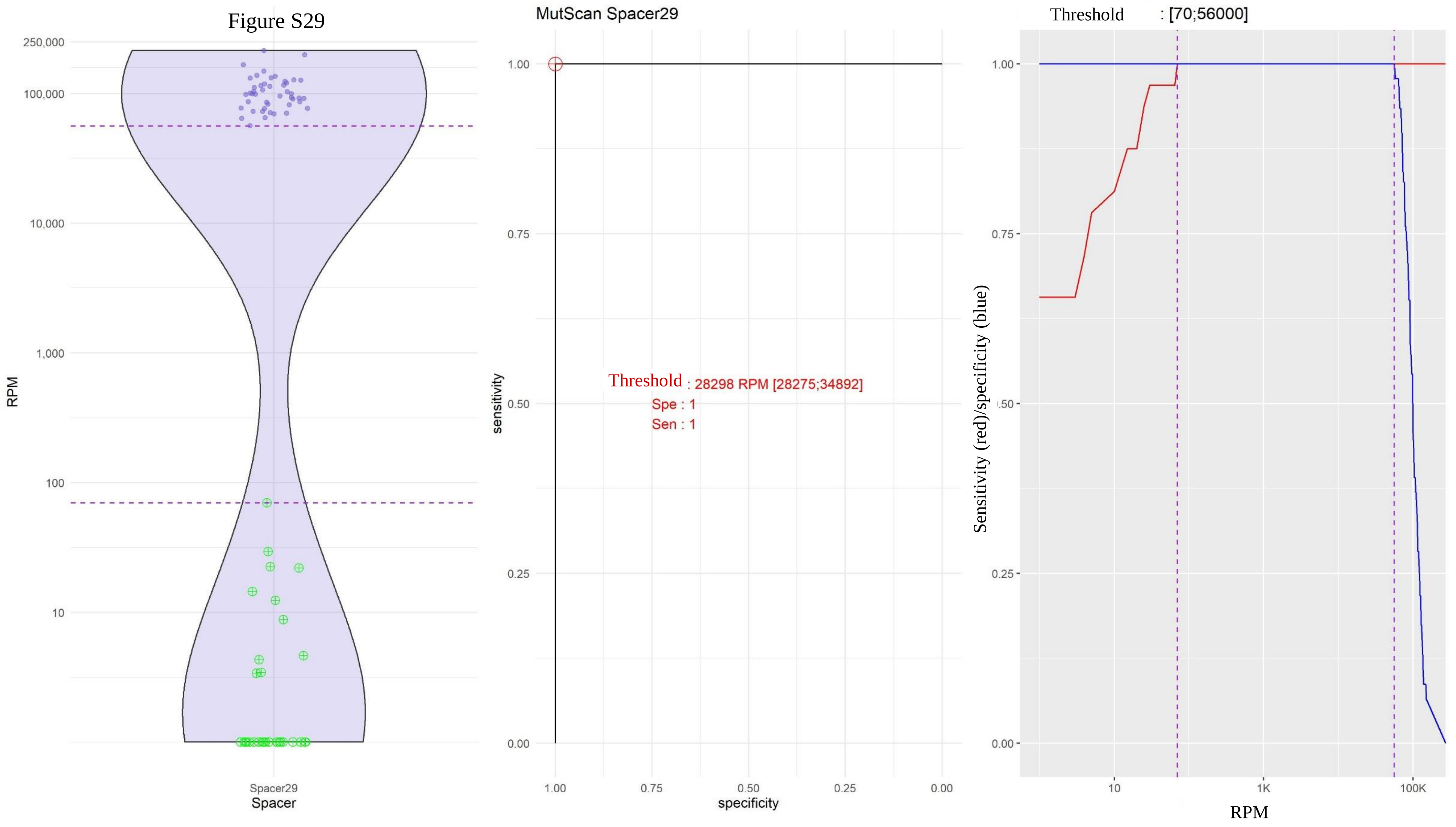
Figure S29
250,000
100,000
10,000
1,000
100
10
RPM
Spacer29
Spacer
MutScan Spacer29
1.00
0.75
0.50
0.25
0.00
sensitivity
specificity
Threshold : 28298 RPM [28275;34892]
Spe : 1
Sen : 1
Threshold : [70;56000]
Sensitivity (red)/specificity (blue)
10
1K
100K
RPM

Figure S30

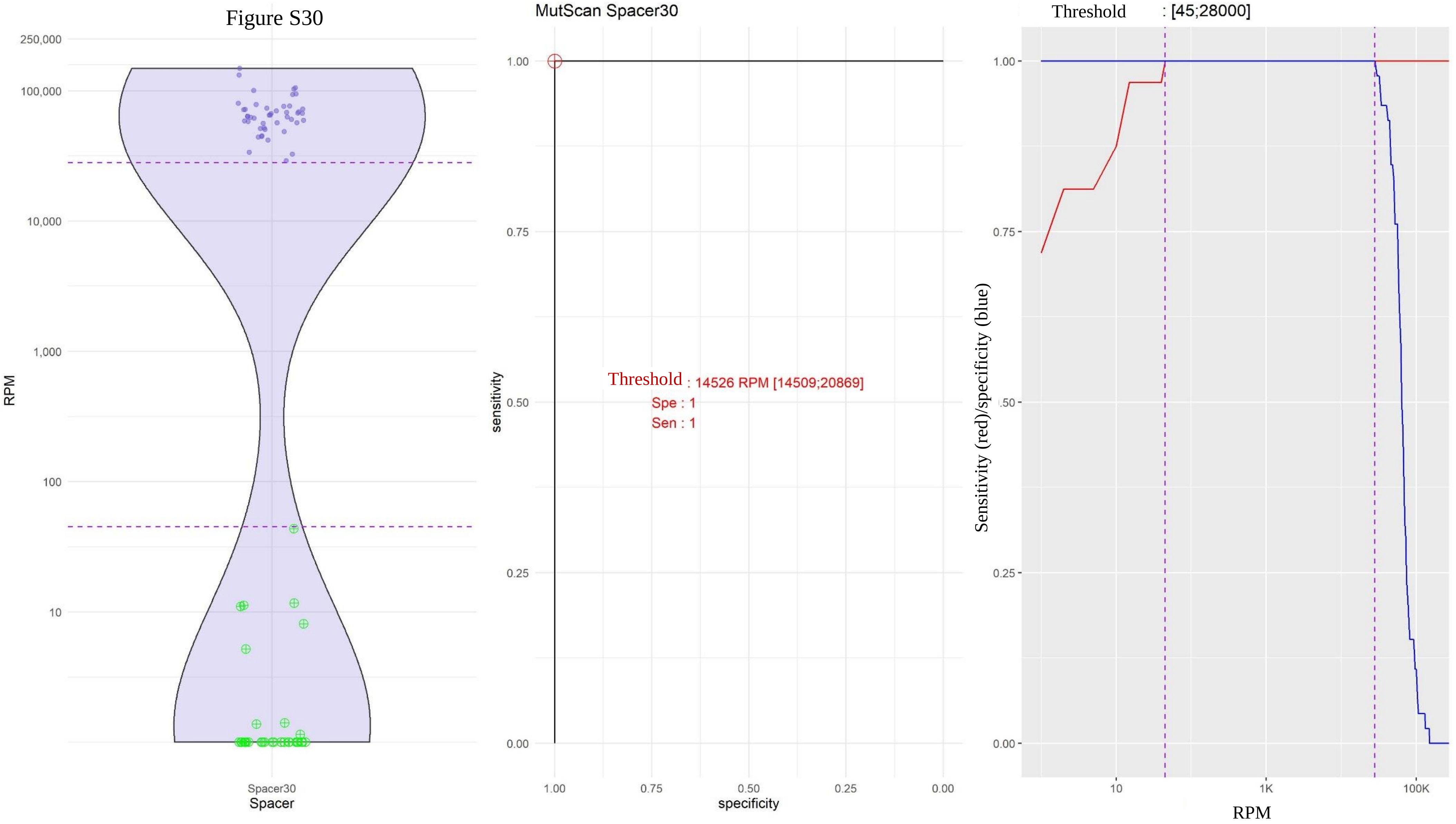

250,000
100,000
10,000
1,000
100
10
RPM
Spacer30
Spacer
MutScan Spacer30
1.00
0.75
0.50
0.25
0.00
sensitivity
specificity
Threshold : 14526 RPM [14509;20869]
Spe : 1
Sen : 1
Threshold : [45;28000]
Sensitivity (red)/specificity (blue)
10
1K
100K
RPM

Figure S31

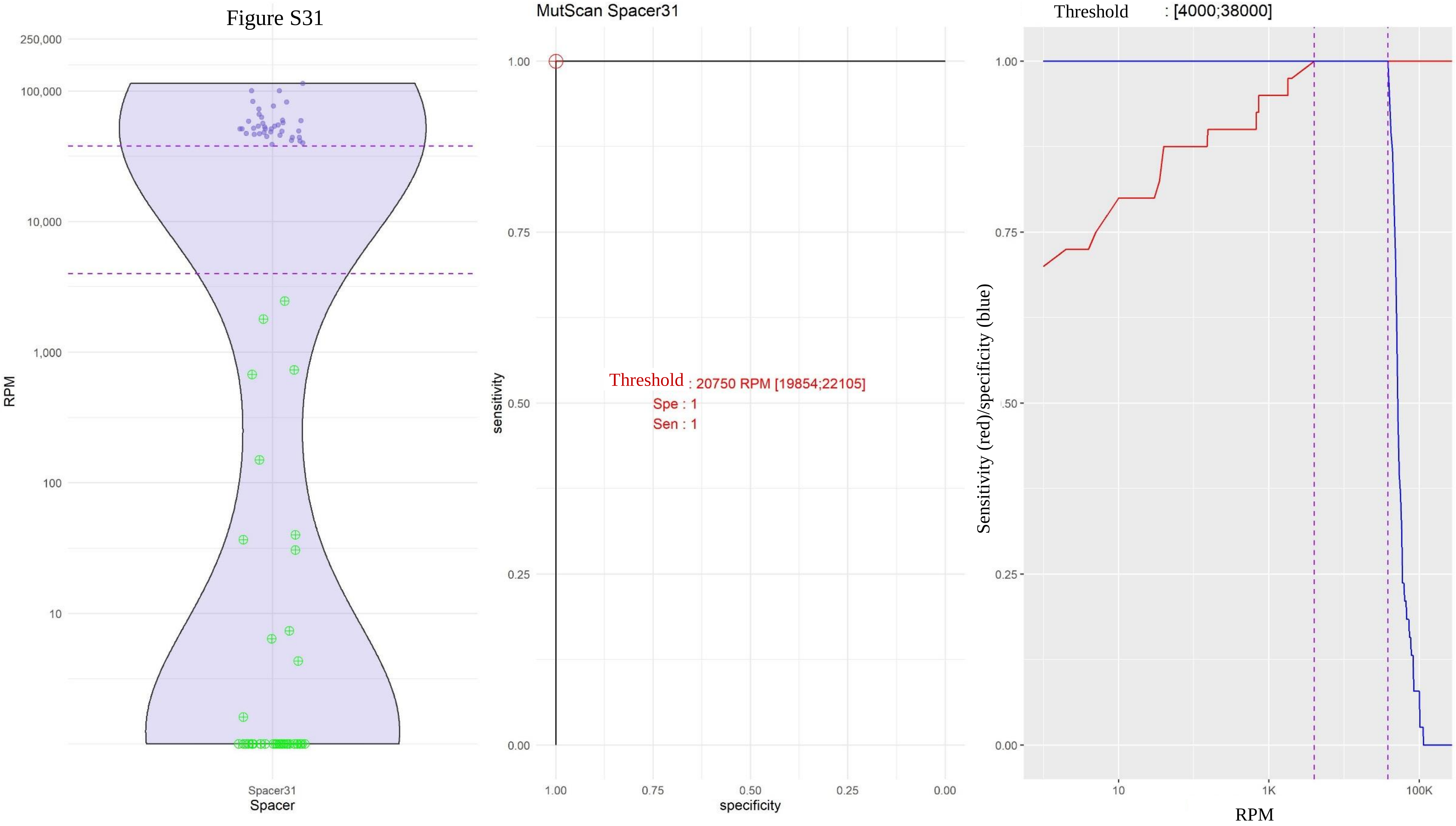

250,000
100,000
10,000
1,000
100
10
RPM
Spacer31
Spacer
MutScan Spacer31
1.00
0.75
0.50
0.25
0.00
sensitivity
specificity
Threshold : 20750 RPM [19854;22105]
Spe : 1
Sen : 1
Threshold : [4000;38000]
Sensitivity (red)/specificity (blue)
10
1K
100K
RPM

Figure S32

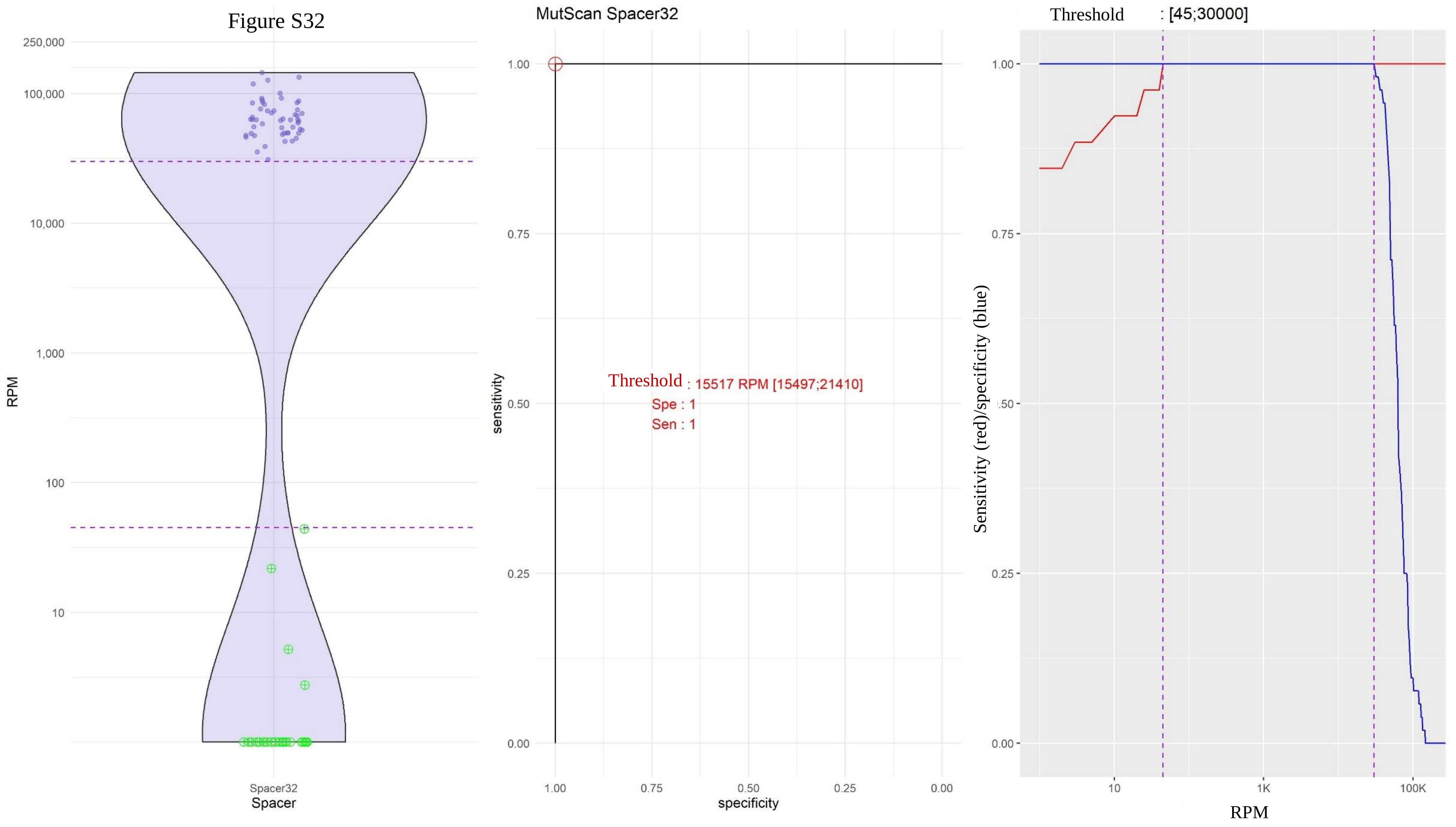
250,000
100,000
10,000
1,000
100
10
RPM
Spacer32
Spacer
MutScan Spacer32
1.00
0.75
0.50
0.25
0.00
sensitivity
specificity
Threshold : 15517 RPM [15497;21410]
Spe : 1
Sen : 1
Threshold : [45;30000]
Sensitivity (red)/specificity (blue)
10
1K
100K
RPM

Figure S33

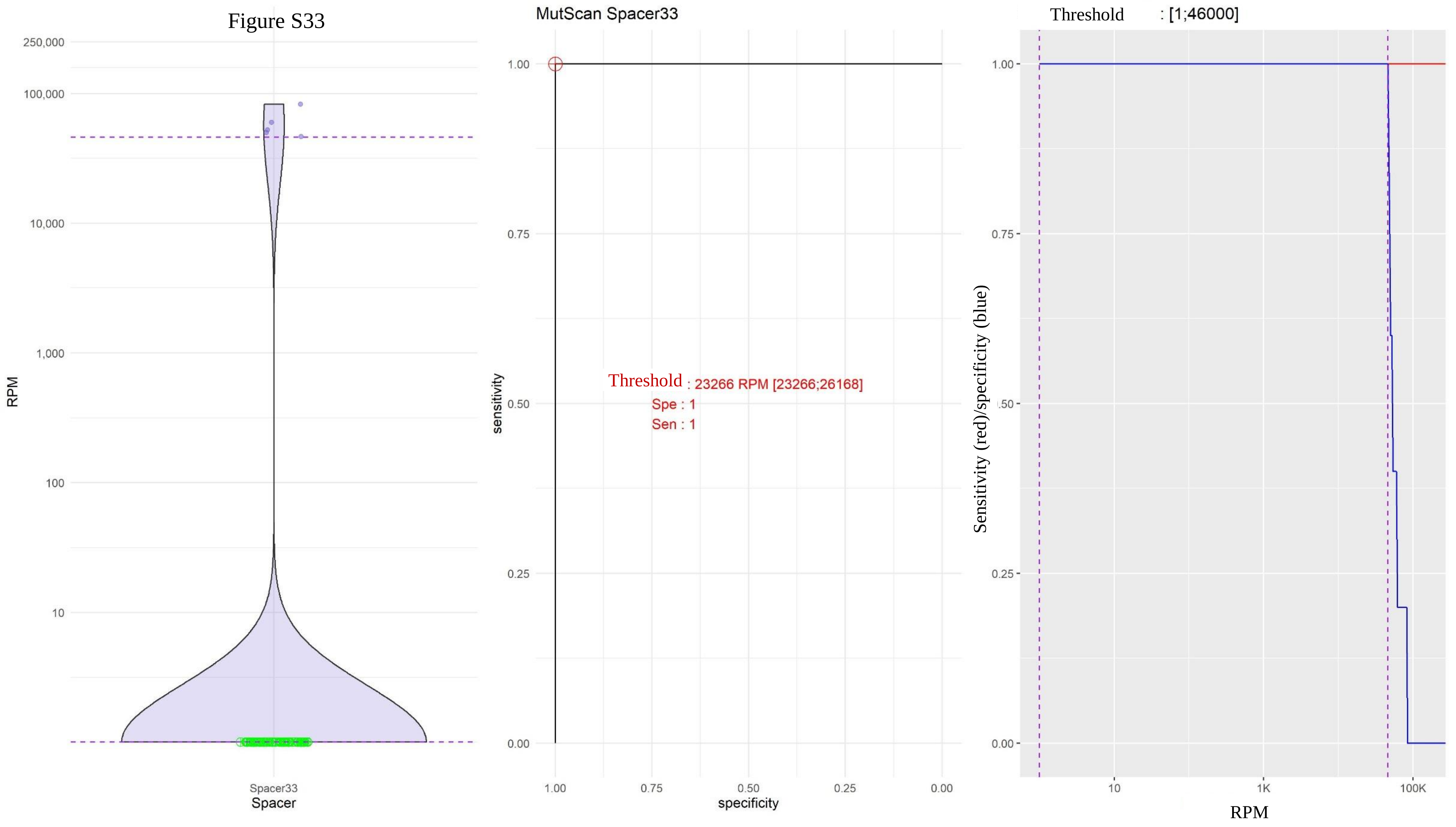

250,000
100,000
10,000
1,000
100
10
RPM
Spacer33
Spacer
MutScan Spacer33
1.00
0.75
0.50
0.25
0.00
sensitivity
specificity
Threshold : 23266 RPM [23266;26168]
Spe : 1
Sen : 1
Threshold : [1;46000]
Sensitivity (red)/specificity (blue)
10
1K
100K
RPM

Figure S34

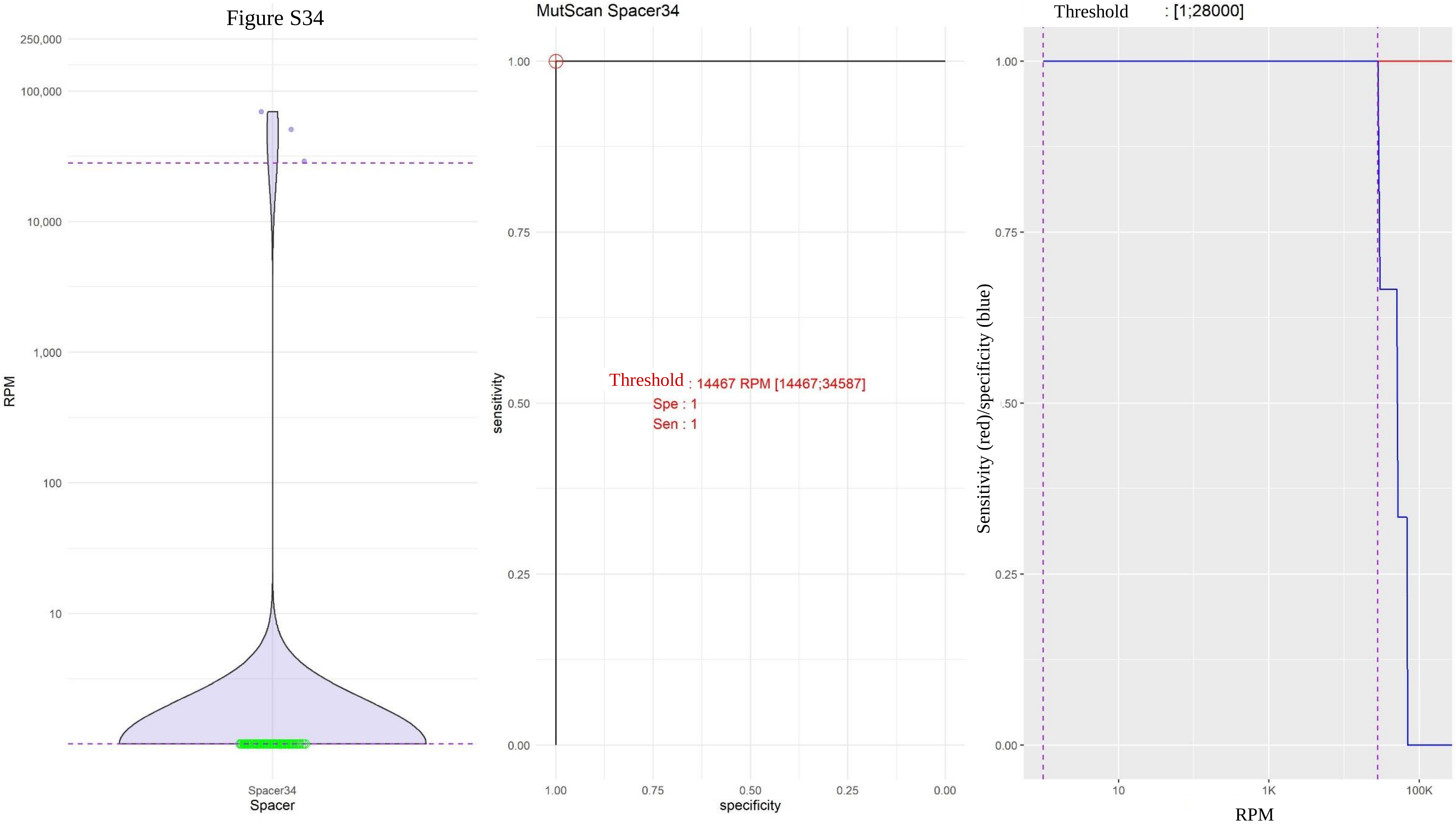

MutScan Spacer34
Threshold : [1;28000]
250,000
100,000
10,000
1,000
100
10
RPM
Spacer34
Spacer
1.00
0.75
0.50
0.25
0.00
sensitivity
specificity
Threshold : 14467 RPM [14467;34587]
Spe : 1
Sen : 1
Sensitivity (red)/specificity (blue)
10
1K
100K
RPM

Figure S35

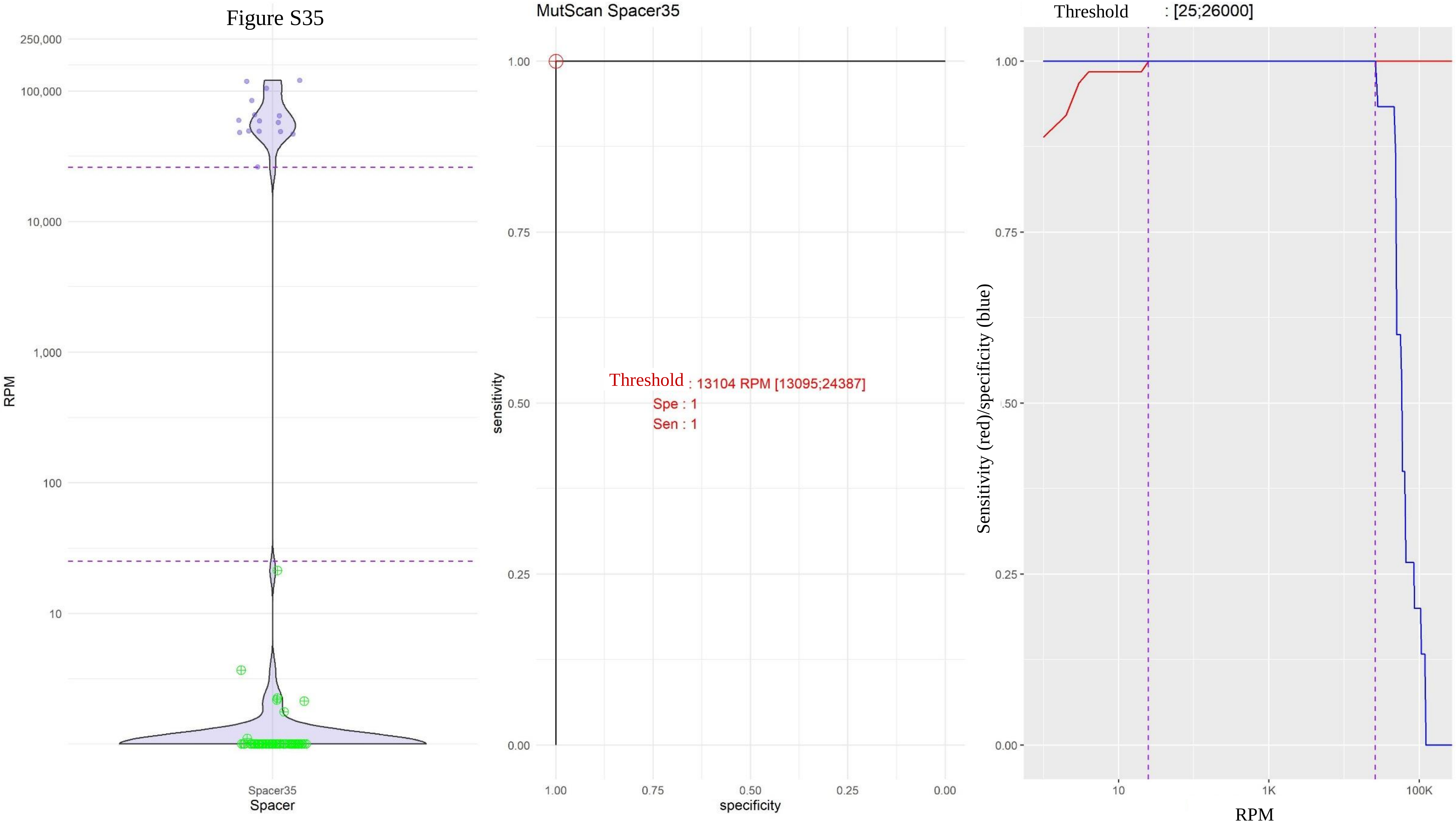

250,000
100,000
10,000
1,000
100
10
RPM
Spacer35
Spacer
MutScan Spacer35
1.00
0.75
0.50
0.25
0.00
sensitivity
specificity
Threshold : 13104 RPM [13095;24387]
Spe : 1
Sen : 1
Threshold : [25;26000]
Sensitivity (red)/specificity (blue)
10
1K
100K
RPM

Figure S36

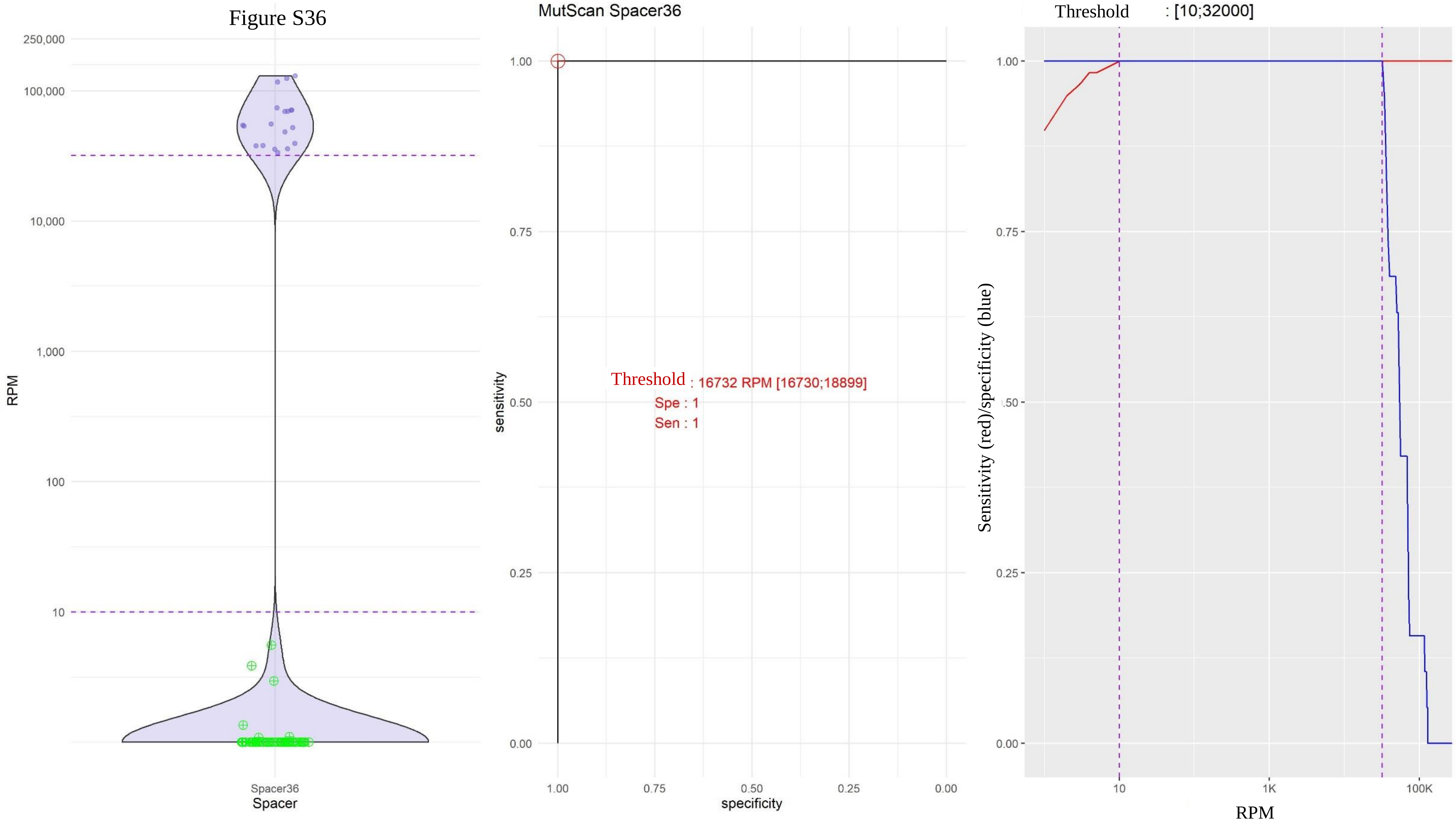
250,000
100,000
10,000
1,000
100
10
RPM
Spacer36
Spacer
MutScan Spacer36
1.00
0.75
0.50
0.25
0.00
sensitivity
specificity
Threshold : 16732 RPM [16730;18899]
Spe : 1
Sen : 1
Threshold : [10;32000]
Sensitivity (red)/specificity (blue)
10
1K
100K
RPM

Figure S37

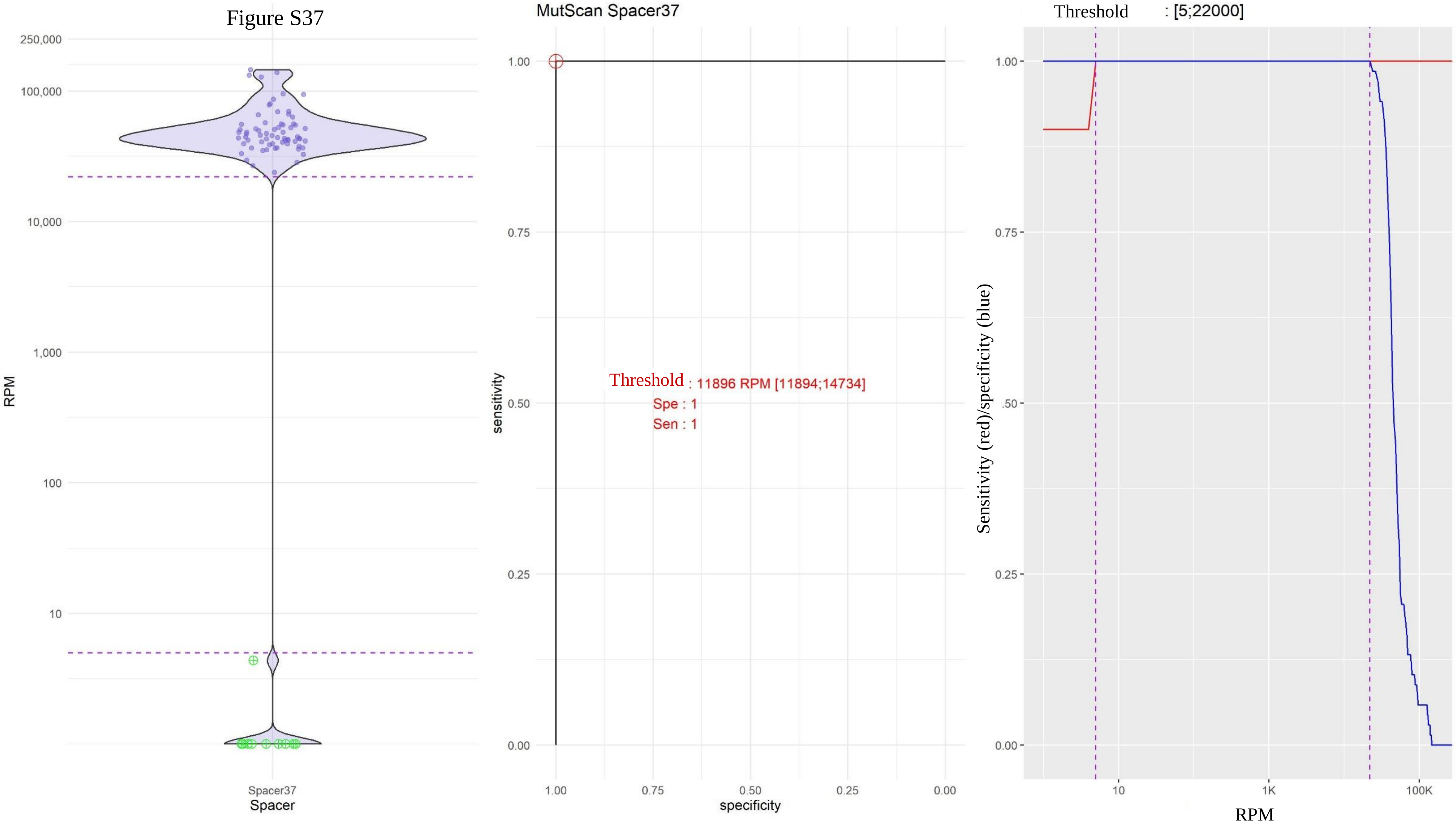
250,000
100,000
10,000
1,000
100
10
RPM
Spacer37
Spacer
MutScan Spacer37
1.00
0.75
0.50
0.25
0.00
sensitivity
specificity
Threshold : 11896 RPM [11894;14734]
Spe : 1
Sen : 1
Threshold : [5;22000]
Sensitivity (red)/specificity (blue)
10
1K
100K
RPM

Figure S38

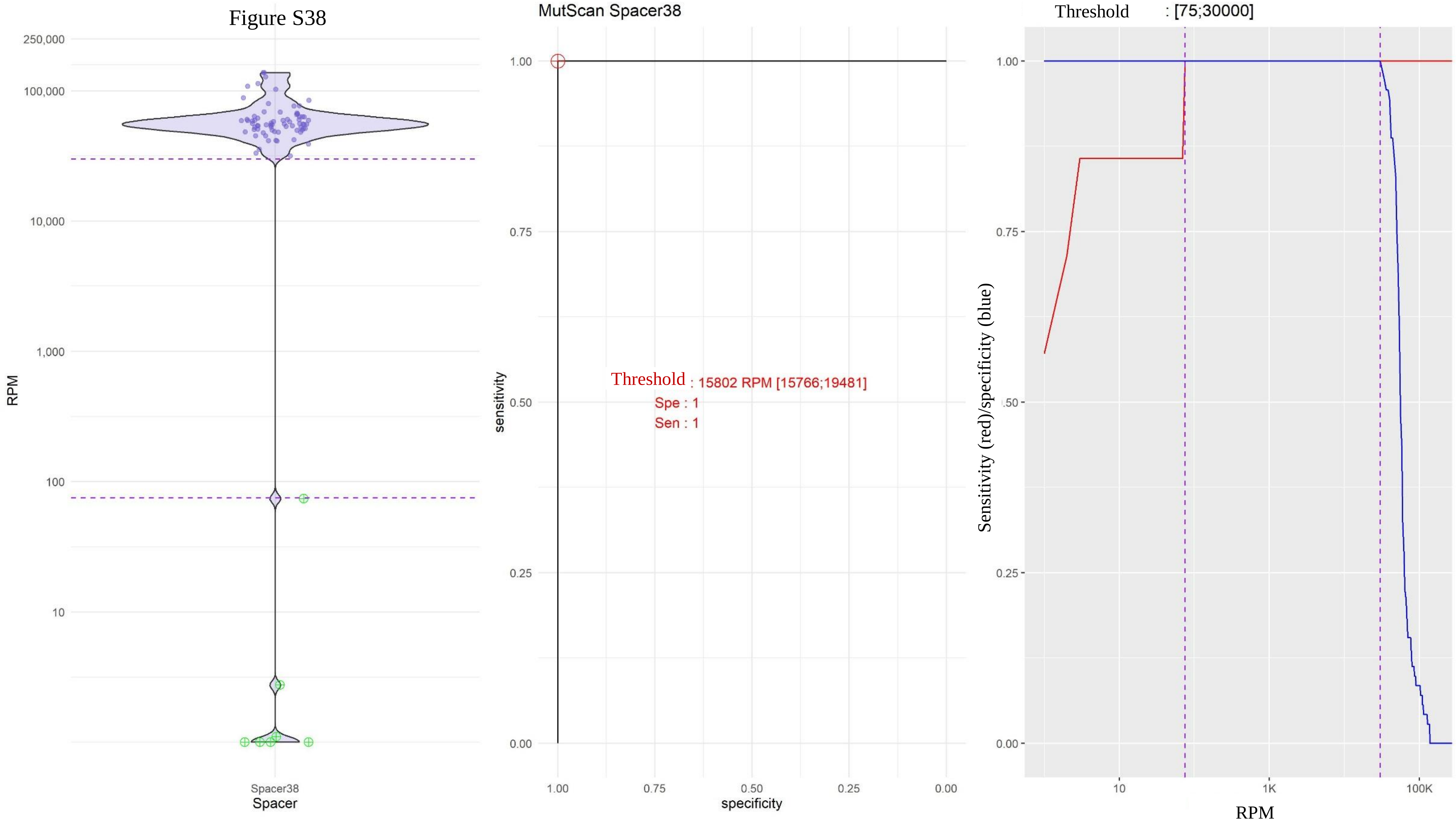

MutScan Spacer38
Threshold : [75;30000]
250,000
100,000
10,000
1,000
100
10
RPM
Spacer38
Spacer
1.00
0.75
0.50
0.25
0.00
sensitivity
specificity
Threshold : 15802 RPM [15766;19481]
Spe : 1
Sen : 1
Sensitivity (red)/specificity (blue)
10
1K
100K
RPM

Figure S39

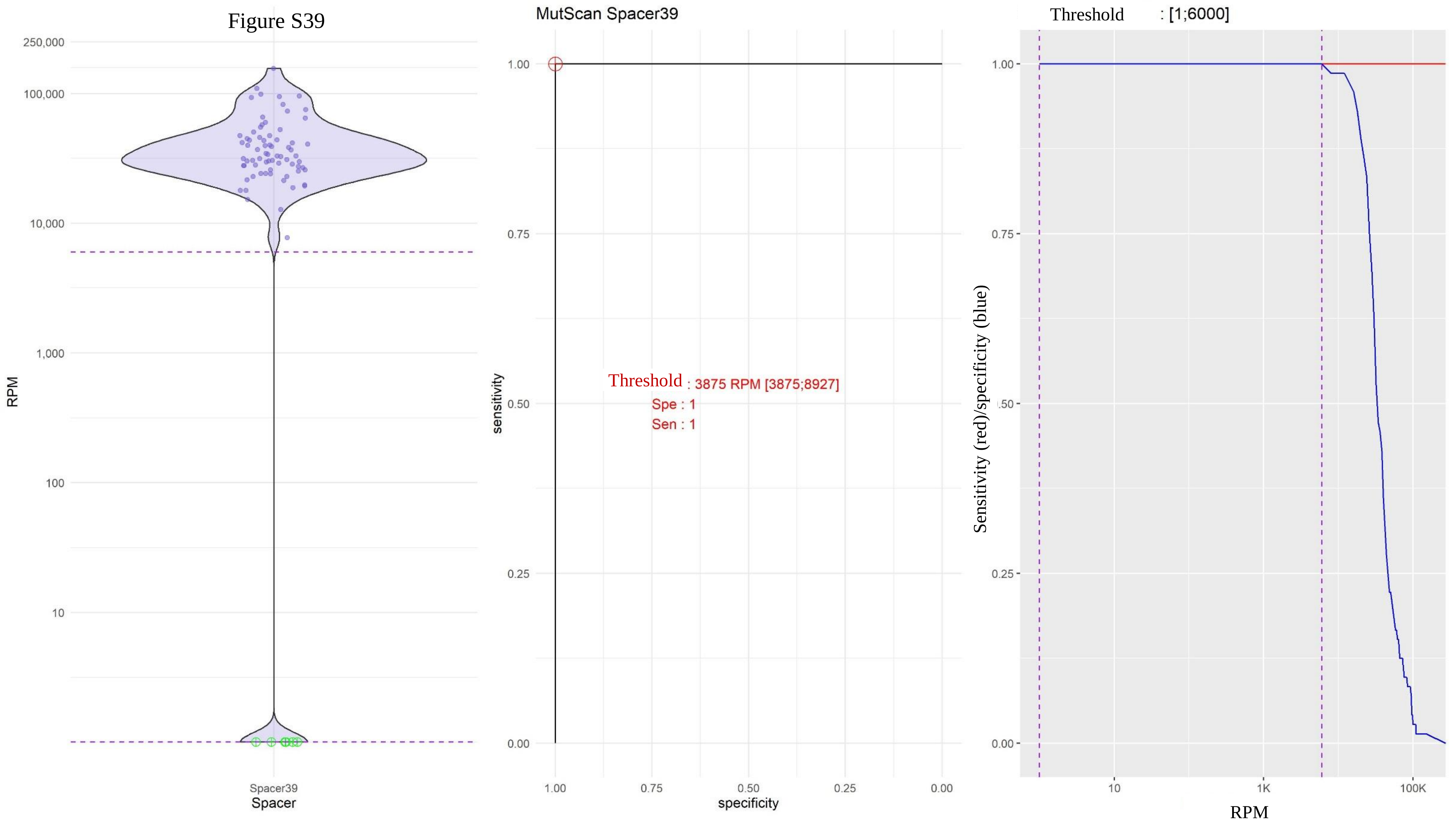
250,000
100,000
10,000
1,000
100
10
RPM
Spacer39
Spacer
MutScan Spacer39
1.00
0.75
0.50
0.25
0.00
sensitivity
specificity
Threshold : 3875 RPM [3875;8927]
Spe : 1
Sen : 1
Threshold : [1;6000]
Sensitivity (red)/specificity (blue)
10
1K
100K
RPM

Figure S40

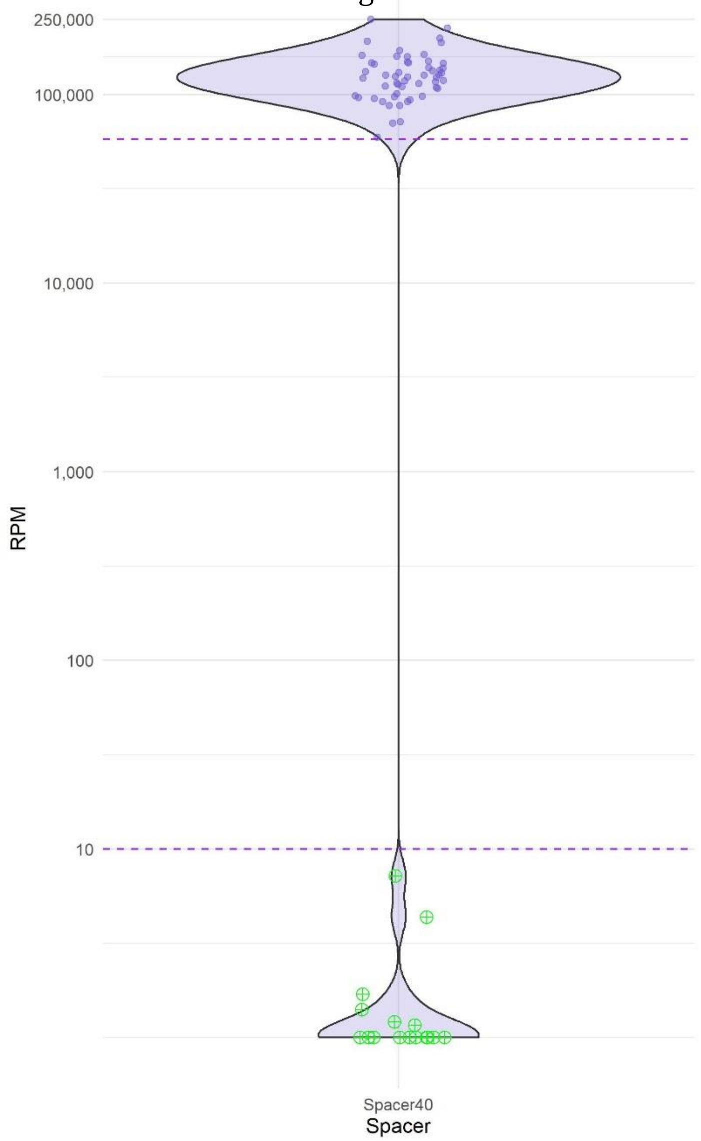

250,000
100,000
10,000
1,000
100
10
RPM
Spacer40
Spacer

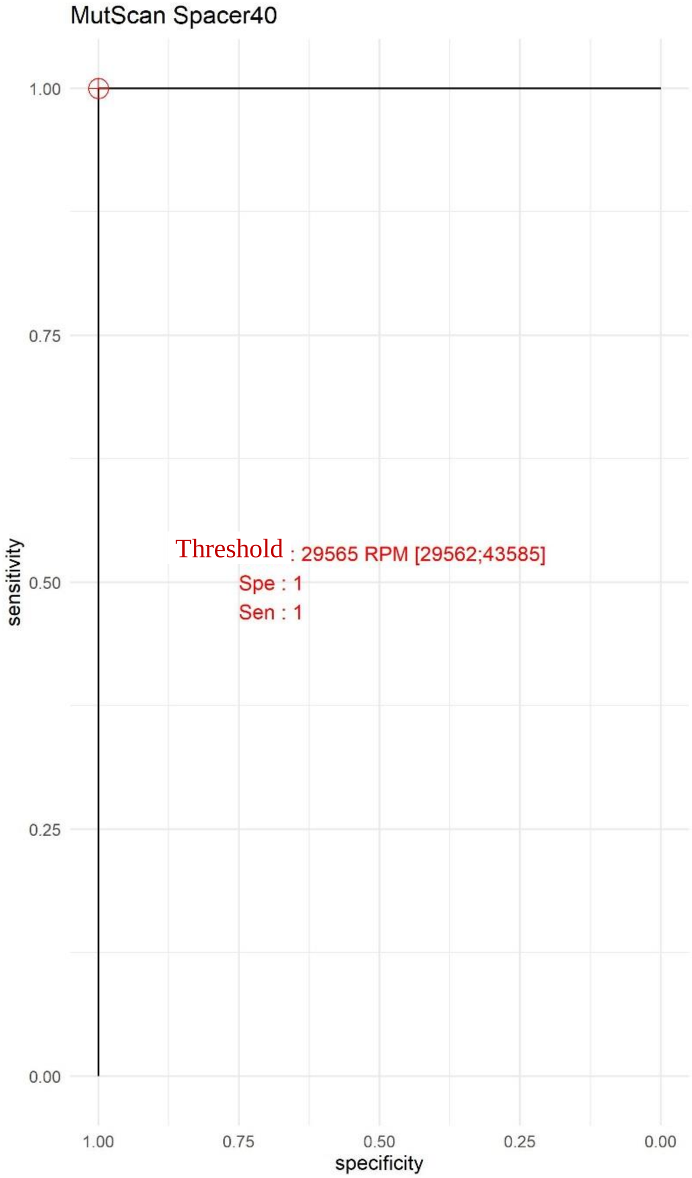

MutScan Spacer40
1.00
0.75
0.50
0.25
0.00
sensitivity
specificity
Threshold : 29565 RPM [29562;43585]
Spe : 1
Sen : 1

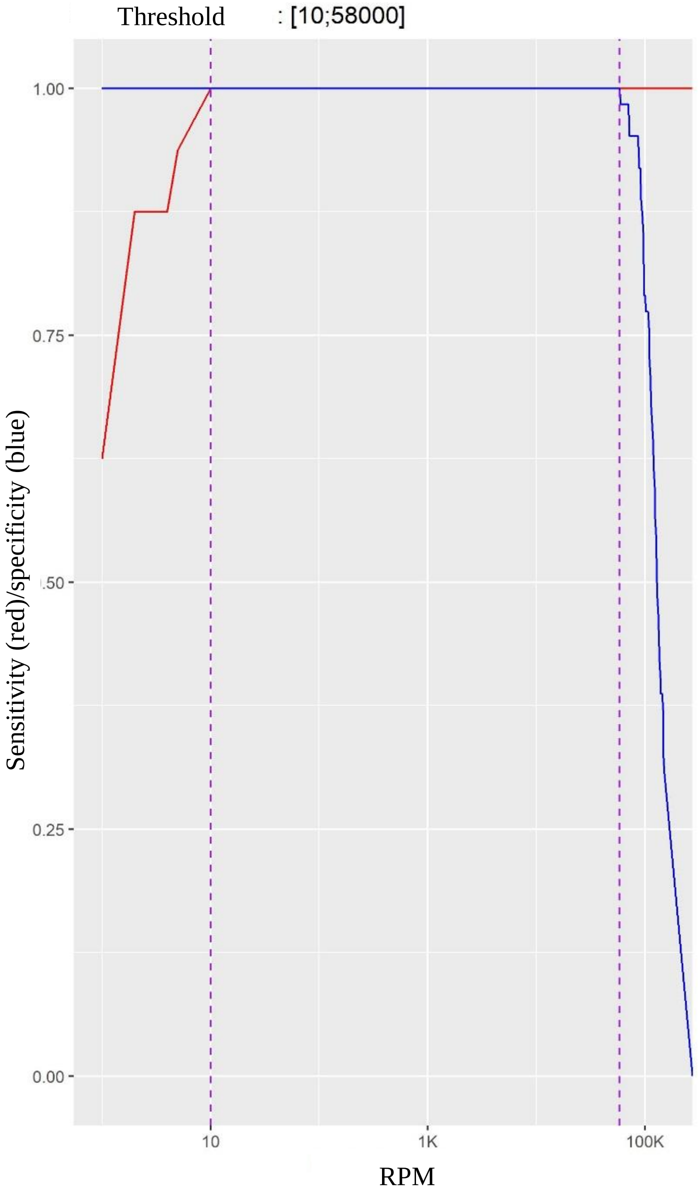

Threshold : [10;58000]
1.00
0.75
.50
0.25
0.00
Sensitivity (red)/specificity (blue)
10
1K
100K
RPM

Figure S41

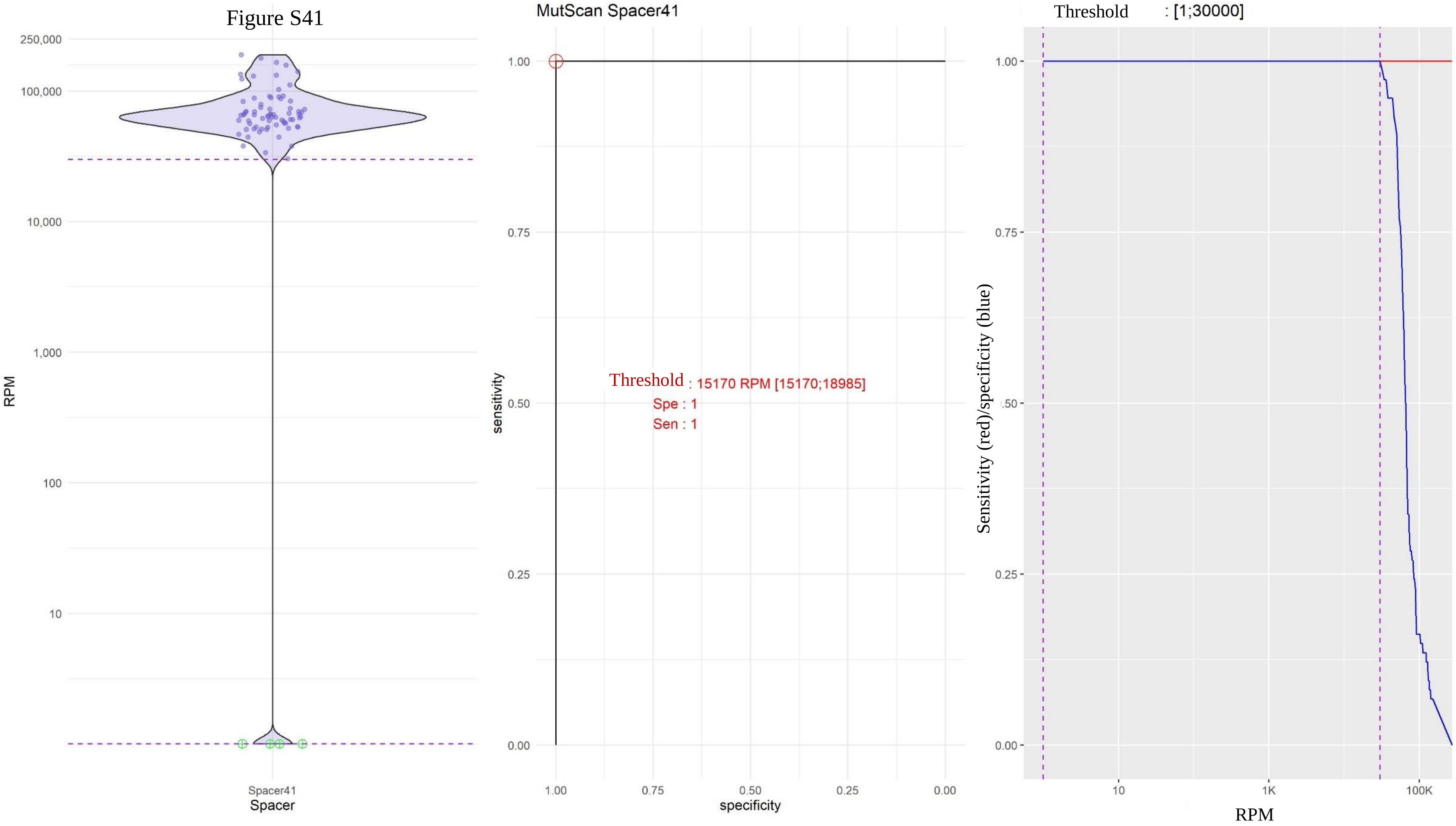
MutScan Spacer41
Threshold : [1;30000]
250,000
100,000
10,000
1,000
100
10
RPM
Spacer41
Spacer
1.00
0.75
0.50
0.25
0.00
sensitivity
specificity
Threshold : 15170 RPM [15170;18985]
Spe : 1
Sen : 1
Sensitivity (red)/specificity (blue)
10
1K
100K
RPM

Figure S42

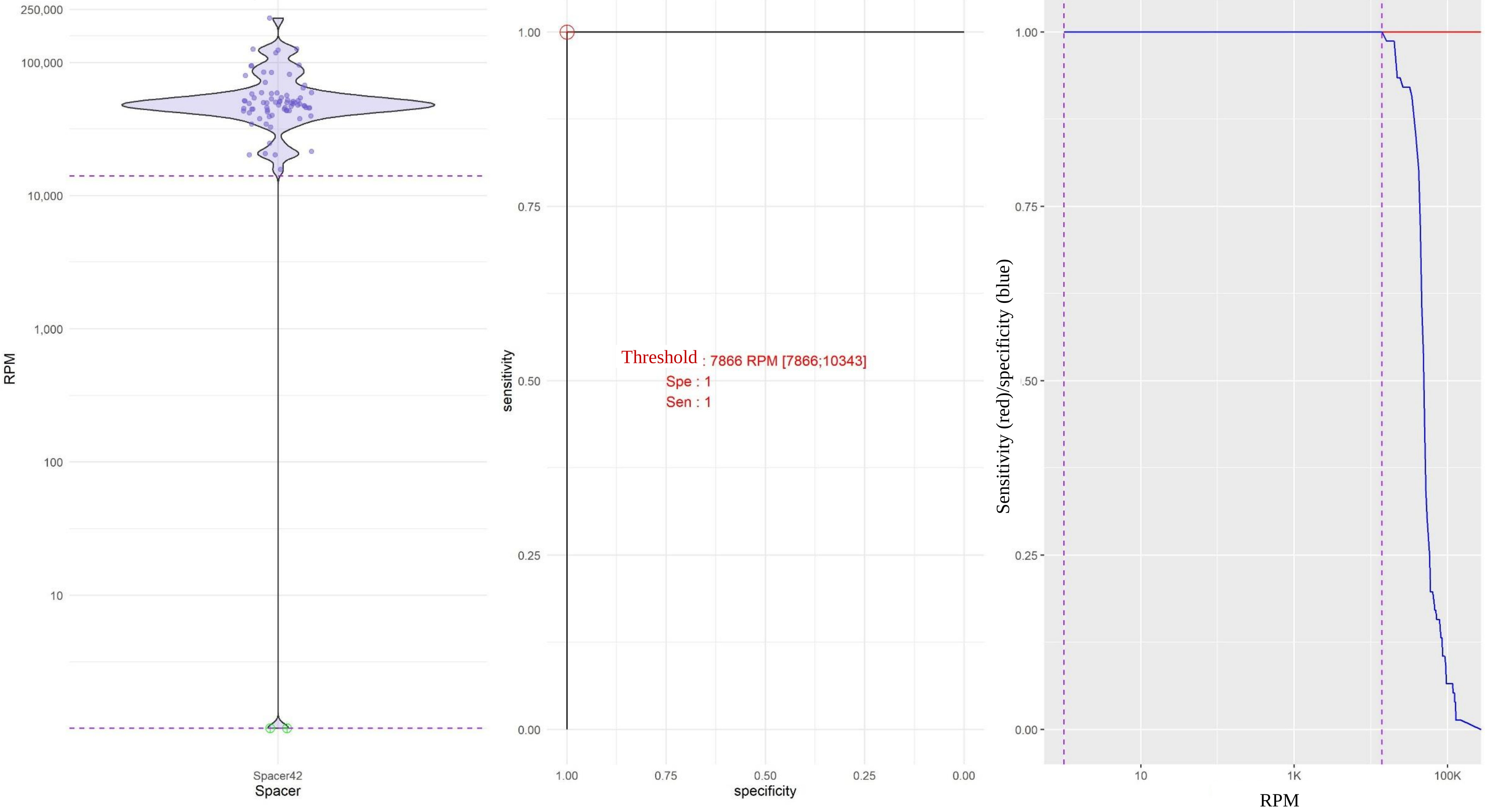

MutScan Spacer42
Threshold : [1;14000]
250,000
100,000
10,000
1,000
100
10
RPM
Spacer42
Spacer
1.00
0.75
0.50
0.25
0.00
sensitivity
specificity
Threshold : 7866 RPM [7866;10343]
Spe : 1
Sen : 1
Sensitivity (red)/specificity (blue)
10
1K
100K
RPM

Figure S43

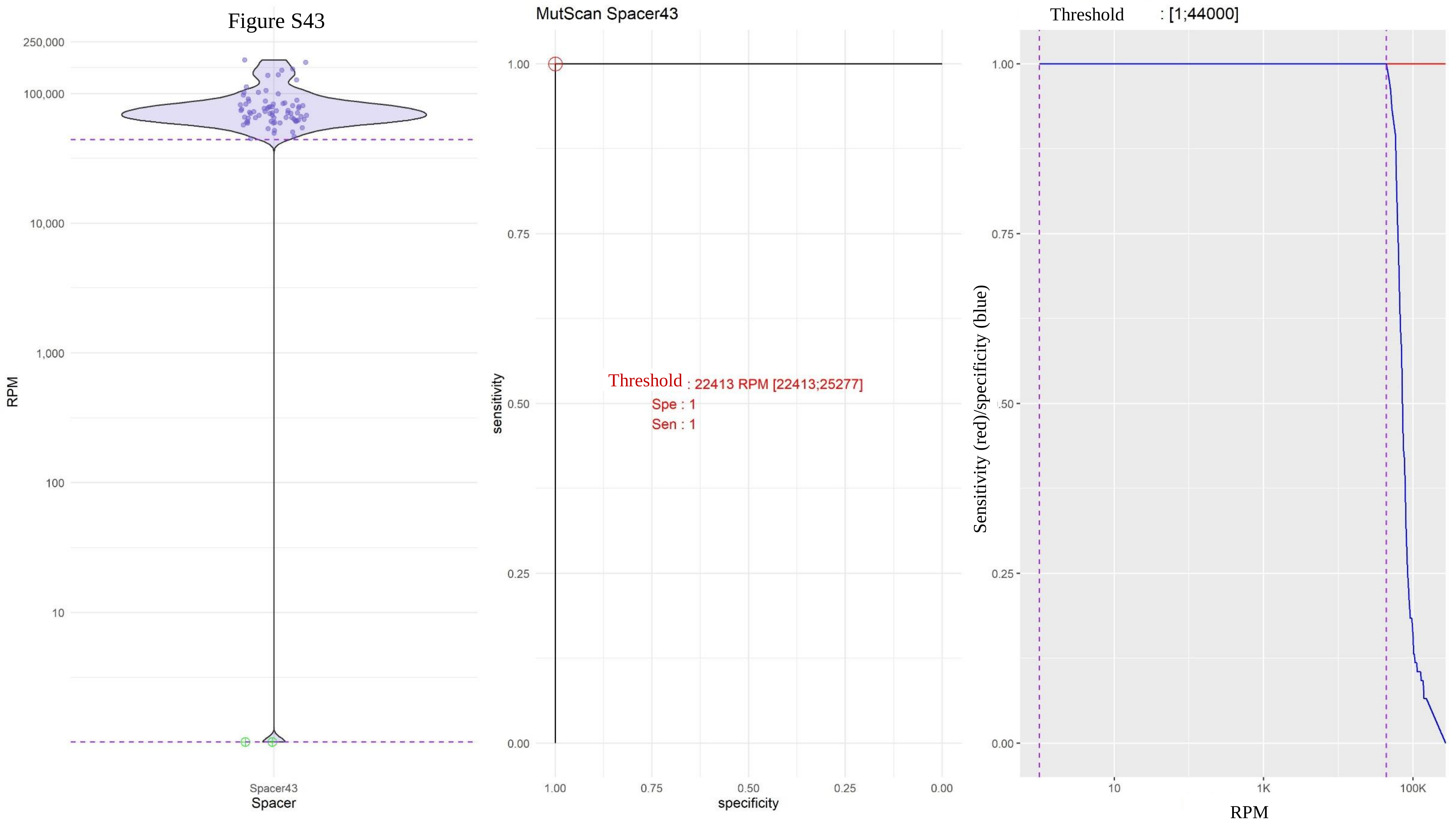

250,000
100,000
10,000
1,000
100
10
RPM
Spacer43
Spacer
MutScan Spacer43
1.00
0.75
0.50
0.25
0.00
sensitivity
specificity
Threshold : 22413 RPM [22413;25277]
Spe : 1
Sen : 1
Threshold : [1;44000]
Sensitivity (red)/specificity (blue)
10
1K
100K
RPM
